# Defining proteoform-specific interactions for drug targeting in a native cell signalling environment

**DOI:** 10.1038/s41557-024-01711-w

**Published:** 2025-01-13

**Authors:** Corinne A. Lutomski, Jack L. Bennett, Tarick J. El-Baba, Di Wu, Joshua D. Hinkle, Sean A. Burnap, Idlir Liko, Christopher Mullen, John E. P. Syka, Weston B. Struwe, Carol V. Robinson

**Affiliations:** 1 Physical and Theoretical Chemistry Laboratory, Department of Chemistry, University of Oxford, Oxford, UK; 2 Kavli Institute for Nanoscience Discovery, University of Oxford, Oxford, UK; 3 Thermo Fisher Scientific, San Jose, CA USA; 4 Department of Biochemistry, University of Oxford, Oxford, UK; 5 OMass Therapeutics, Oxford, UK

**Keywords:** Mass spectrometry, Structural biology

## Abstract

Understanding the dynamics of membrane protein–ligand interactions within a native lipid bilayer is a major goal for drug discovery. Typically, cell-based assays are used, however, they are often blind to the effects of protein modifications. In this study, using the archetypal G protein-coupled receptor rhodopsin, we found that the receptor and its effectors can be released directly from retina rod disc membranes using infrared irradiation in a mass spectrometer. Subsequent isolation and dissociation by infrared multiphoton dissociation enabled the sequencing of individual retina proteoforms. Specifically, we categorized distinct proteoforms of rhodopsin, localized labile palmitoylations, discovered a Gβγ proteoform that abolishes membrane association and defined lipid modifications on G proteins that influence their assembly. Given reports of undesirable side-effects involving vision, we characterized the off-target drug binding of two phosphodiesterase 5 inhibitors, vardenafil and sildenafil, to the retina rod phosphodiesterase 6 (PDE6). The results demonstrate differential off-target reactivity with PDE6 and an interaction preference for lipidated proteoforms of G proteins. In summary, this study highlights the opportunities for probing proteoform–ligand interactions within natural membrane environments.

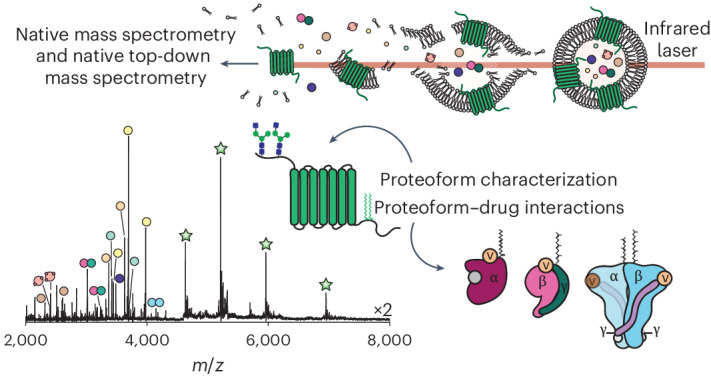

## Abstract (web summary)

G protein-coupled receptors and their effectors can now be released directly from a lipid bilayer using infrared irradiation for proteoform-level characterization by native top-down mass spectrometry. This represents a critical development for drug discovery, as the direct role of post-translational modifications in protein–protein and protein–drug interactions can be characterized.

## Main

Alternative splicing and post-translational modifications (PTMs) alter the molecular identity of proteins, yielding hundreds of thousands of unique human ‘proteoforms’ from only ~20,000 protein-coding genes^1^. Proteoforms have been extensively catalogued in various cell types using large-scale proteomics, uncovering tens of thousands of distinct proteins in an attempt to link molecular biology to a phenotypic presentation^2–6^. Such studies have become increasingly important for the development of new therapeutics, as targeting unique proteoforms promises personalized therapies with fewer off-target effects^7^. For example, the pharmaceutical modulation of protein modifications using small-molecule inhibitors of protein kinases^8^, deacetyltransferases^9,10^ and farnesyltransferases^11^ has been found to enable the effective treatment of many cancers. However, the massive diversity and complexity of human proteoforms in vivo challenges many screening approaches that aim to rationally achieve proteoform-specific modulation. Deciphering the direct effects of PTMs on protein interactions within their native biological environment therefore represents a critical challenge in the development of safe and effective drugs.

Mass spectrometry (MS)-based proteomics is the contemporary method of choice for the characterization of proteoforms and their remodelling in the context of disease^12,13^. Typically, proteins from cell or tissue lysates are proteolysed into peptides, which are then separated by liquid chromatography (LC) and identified by tandem MS (MS/MS) in a process known as bottom-up proteomics. Statistical methods can use database searching to assemble the set of identified peptides into the antecedent proteins. However, peptides can be shared across many proteoforms, such that the direct link between the uniquely modified peptide and the original modified protein is lost^14^. To preserve this link, top-down proteomics omits protease digestion and measures intact proteoforms, avoiding the need for inference. This approach allows for clear identification of PTMs on a single protein, including membrane proteins, but still requires fractionation via denaturing LC before MS/MS analysis^15–20^. Thus, the critical link between PTMs and their direct role in protein interactions is severed.

Native MS reveals protein interactions through their direct analysis in a mass spectrometer. Meanwhile, native top-down MS is an emerging technique in which proteoforms can be characterized within complexes, thereby directly linking PTMs to their involvement in protein interactions^21–24^. Membrane proteins, which represent >60% of potential drug targets^25^, require prior stabilization in detergents or membrane mimetics. These detergents or mimetics remain associated with the protein during transit into the gas phase and need to be removed before mass analysis. Typically, collisional activation or infrared laser irradiation^26–28^ has been used to liberate membrane proteins from such mimetics. Historically, this has been limited to artificial mimetics^29^; only recently has it been possible to study membrane protein complexes ejected directly from their native lipid bilayers^30,31^. This finding was controversial at the time^32^, as native top-down sequencing for the identification of proteins liberated directly from the lipid bilayer was not yet possible with the available instrumentation. Liberating proteins from the lipid bilayer required maximum acceleration potentials in dedicated collision cells, thereby preventing fragmentation for identification. Protein assignment relied on matching cofactor binding data and intact mass measurements with proteoform databases. Sequencing native membrane proteins from endogenous lipid bilayers to discover proteoforms required significant technological innovation.

In this study, overcoming previous limitations, we found that by directing a 10.6 µm wavelength CO_2_ laser into a high-pressure cell of a linear ion trap we could achieve (1) controlled liberation of membrane proteins and membrane-associated proteins from native membranes, and (2) native top-down sequencing of the liberated protein complexes and their proteoforms by infrared multiphoton dissociation (IRMPD). Using this approach, we were able to identify the fine molecular details of different proteoforms present in a heterogeneous milieu. We selected rod outer disc membranes of photoreceptors for our investigation as rhodopsin phototransduction is well characterized: the photon-induced signalling cascade is tightly regulated by PTMs that control dynamic protein–protein and protein–nucleotide interactions within signalling effectors such as G proteins and rod phosphodiesterase 6 (PDE6)^33^. The results show that IRMPD provides adequate sequence coverage for high-confidence protein identification and also preserves labile lipid modifications such as palmitoylation, farnesylation, geranyl-geranylation and polyunsaturated myristoylation. Having achieved this in-depth characterization of modified retina proteins, we then challenged the system with PDE inhibitors capable of crossing the blood–retina barrier. We elucidated the off-target binding of PDE5 inhibitors to retinal PDE6 and found evidence for additional off-target drug binding to G proteins with hydrophobic PTMs. Overall, we found that we can locate even labile PTMs, identify proteoforms and define their interactions, as well as modulate the native membrane environment with established drugs to discover previously uncharacterized off-target interactions.

## Results

### Liberating membrane and intradiscal proteins

**Fig. 1:**
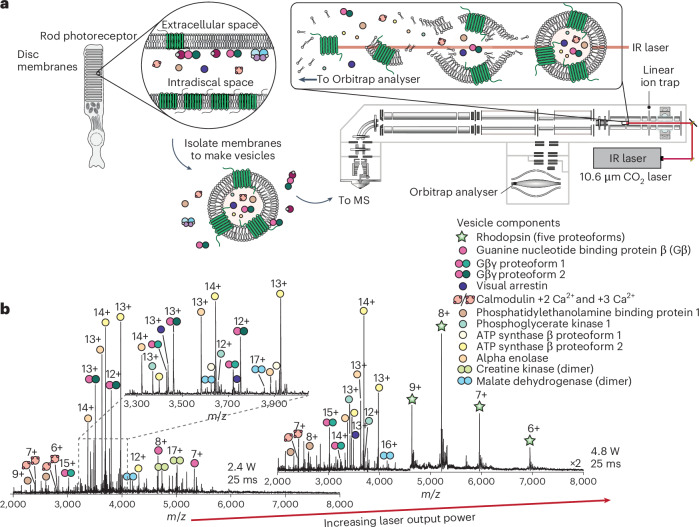
Liberating membrane and intradiscal proteins from vesicles derived from an endogenous lipid bilayer. **a**, Disc membranes were isolated and disrupted via sonication to form vesicles. The vesicles were introduced directly into a mass spectrometer modified with an infrared (IR) laser directed into the high-pressure cell of a linear ion trap analyser. **b**, Left: mass spectrum obtained using a laser output power of 2.4 W with an irradiation time of 25 ms. Adjacent charge state series are denoted by circles. Inset: densely populated region of the mass spectrum. Right: at an increased laser output power of 4.8 W, a protein distribution corresponding to rhodopsin (denoted by stars) emerges at *m*/z > 4,500 (magnified ×2) as the membrane protein has been liberated from the native lipid bilayer. The identities of all proteins were confirmed using IRMPD and are listed in the legend.

We first explored the use of infrared photons to controllably release proteins from lipid vesicles in a mass spectrometer (Fig. 1a). For a fixed irradiation time of 25 ms, the mass spectra were markedly different at discrete laser output powers (~2.4 W versus ~4.8 W, or 4% and 8% of the nominal maximum laser output power; Fig. 1b). At the lower laser output power, three abundant protein distributions dominated above many relatively low abundance peaks. Thirteen unique protein charge state distributions were observed in total (Fig. 1b). Increasing the laser output power resulted in the appearance of a new charge state distribution at *m*/*z* > 4,500. This peak series was only observed within a narrow range of laser output powers (~4.8 W to ~5.4 W; Supplementary Fig. 1) and was tentatively assigned to rhodopsin with a measured mass of 41,716 Da. Additional satellite features were also apparent, indicating the presence of at least five possible proteoforms of rhodopsin^31,34^.

Considering the disparate spectra recorded at different infrared laser powers, we hypothesize that, up to 4.8 W, laser irradiation disperses the lipid bilayer, releasing proteins trapped within the core, as well as those associated with the membrane (Fig. 1a, inset). During this process, non-covalent interactions remain intact. Meanwhile, at higher laser outputs (>4.8 W), the energy deposition is sufficiently high that the lipid bilayer dissipates, liberating intact membrane proteins for MS analysis. Thus, for vesicles derived from native membranes, irradiation with infrared photons presents a unique opportunity for tuneable and controlled release of proteins and complexes. These complexes include both soluble and membrane-embedded proteins that are primed for interrogation using top-down MS.

To identify the molecular composition of the most abundant peaks in the spectrum, we isolated by *m*/*z* each charge state within a distribution for subsequent fragmentation. The most abundant peaks in the MS^1^ spectrum were identified as key proteins involved in glycolysis: phosphoglycerate kinase, alpha enolase and the ATP synthase subunit beta. Additional low abundant peaks corresponded to malate dehydrogenase dimers and creatine kinase. These proteins are highly abundant in cells and are likely to originate from co-fractionating mitochondria that support the metabolic demand of the retina^35^. Evidence for modifications to these proteins, which caused them to differ from the canonical amino acid sequences, was found in the fragmentation spectra (Supplementary Table 1 and Supplementary Figs. 2–5).

**Extended Data Fig. 1:**
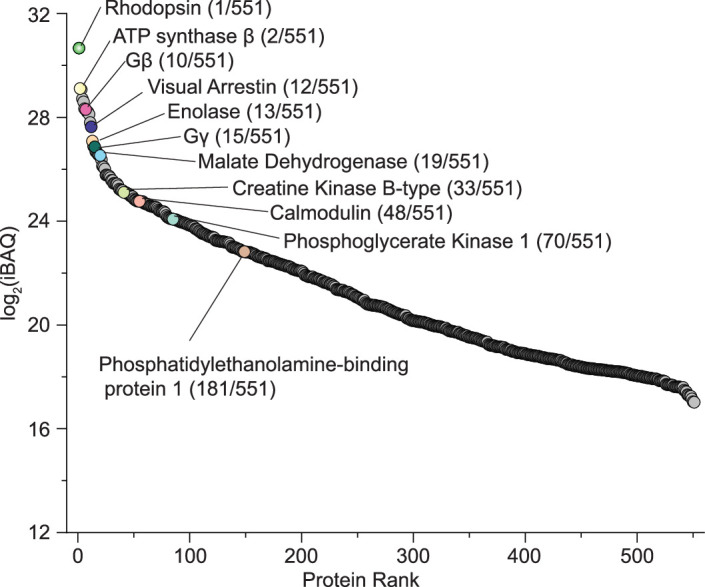
Bottom-up proteomics analysis of rod disc membranes. Plot of each protein ranked by log_2_ of the intensity-based absolute quantification (iBAQ) value. The proteins identified by native MS and top-down MS are annotated with their rank order. Source data

Turning to the remaining proteins, subsequent isolation and fragmentation of individual peaks revealed proteins directly involved in the visual phototransduction cascade. For example, fragmentation of the 13+ ion at *m*/*z* = 3,486 identified the full-length isoform of visual arrestin (S-arrestin) with amino-terminal acetylation (Supplementary Fig. 6). Other peaks in the MS^1^ spectrum were identified as calmodulin (CAM), phosphatidylethanolamine-binding protein 1 (PEBP1) and guanine nucleotide-binding proteins (for example, Gβγ heterodimers), all of which are dynamically involved in protein–protein interactions during phototransduction. The proteins identified by top-down MS were also found by bottom-up MS (Extended Data Fig. 1). Many of the proteins detected by native MS were among the top ~20% most abundant, while others, such as PEBP1, were less abundant but still readily detected. This demonstrates that although native MS surveys only a fraction of the total proteins, it offers a unique opportunity to characterize low-abundance proteoforms in their native context.

### Native top-down sequencing of rhodopsin proteoforms

**Fig. 2:**
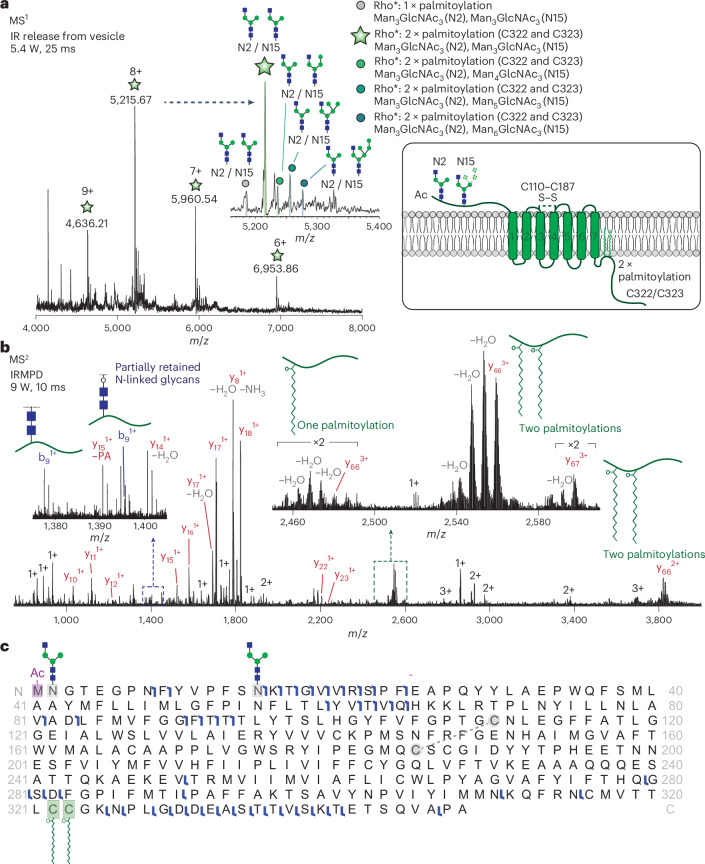
Native top-down sequencing of rhodopsin following liberation from the lipid bilayer. **a**, Native mass spectrum of rhodopsin released from the lipid bilayer. Inset: magnification of the 8+ charge state, revealing five different rhodopsin proteoforms. **b**, MS^2^ spectrum following isolation of the peak at *m*/*z* = 5,215 with an isolation width of 50 *m*/*z* and subjected to IRMPD (9 W, 10 ms irradiation time). b- and y-type fragment ions were matched to a predicted MS^2^ spectrum. Insets: fragment ions indicating the presence of PTMs, including glycosylation, and the likely sites of palmitoylation. **c**, Graphical sequence map of the entire repertoire of fragment ions that were matched to those predicted for the unmodified and modified forms of rhodopsin. A sequence coverage of 14% was obtained.

The proteoform heterogeneity of endogenous rhodopsin was immediately apparent from the mass spectrum (Fig. 2a). The PTMs involved in the activation and recovery stages of rhodopsin signalling are well annotated, and their effects on protein function have been studied using carefully designed mutants^36–39^. We were interested to explore how these PTMs were arranged on individual proteoforms within the native lipid bilayer. A detailed analysis of the 8+ charge state revealed five satellite peaks corresponding to deconvoluted masses ranging from 41.4 kDa to 42.3 kDa (Supplementary Table 1). As the sequence mass of rhodopsin is 39.0 kDa, the higher measured molecular weights (2.4–3.3 kDa) must be due to the contribution of multiple PTMs. The following modifications have been reported: acetylation of Met1, N-linked glycosylation at Asn2 and Asn15, palmitoylation at Cys322 and Cys323, phosphorylation at seven potential sites (Ser334, Thr335, Thr336, Ser338, Thr340, Thr342 and Ser343) and a disulfide bond Cys110–Cys187 linking extracellular loops 1 and 2. Furthermore, the 11-*cis*-retinylidene chromophore, which activates the G protein-coupled receptor (GPCR), interacts through a reversible covalent attachment to the protein at Leu296. Upon photoactivation, 11-*cis*-retinylidene rapidly isomerizes to all-*trans*-retinylidene, followed by hydrolysis and chromophore release, which differentiates rhodopsin from its activated form, Rho* (ref. ^40^). The possible combinations of PTMs and covalent modifications that can be summed to make up the measured intact masses are therefore vast.

To characterize these PTMs definitively, we isolated the 8+ charge states (*m*/*z* = 5,215 with an isolation width of 50 *m*/*z*), which encompassed all proteoforms present within the mass range of ~41.5–41.9 kDa. Subjecting the proteoforms to IRMPD, we obtained fragment ions (Fig. 2b), the most abundant of which were assigned as y-type ions (y_10_^1+^ to y_23_^1+^) originating from backbone cleavages within the flexible carboxy-terminal tail. A high-molecular-weight fragment series (*m*/*z* > 2,400) revealed a cluster of triply charged fragments (y_66_^2+^ and y_67_^2+^) that originated from backbone cleavages in extracellular loop 3. These cleavages liberated large C-terminal fragments containing one or two palmitate modifications at Cys322 and Cys323 that surprisingly survived the fragmentation process (Fig. 2b, inset). The same backbone cleavage products were also present as doubly charged ions (*m*/*z* ~3,800), with the palmitate modifications maintained. It is noteworthy that the palmitates remained attached to the cysteine side chains as this labile modification is typically lost during conventional collision-induced fragmentation processes^41^.

Despite achieving good sequence coverage of the C-terminal domain, the presence of hybrid N-linked glycans at Asn2 and Asn15 precluded a straightforward assignment of b-type fragments originating from the N-terminal domain. As glycosidic bonds are relatively labile compared with the amide bonds of a protein backbone^42^, concomitant glycan and protein fragmentation increases the number of possible sequence ions. We therefore searched for sequence ions habouring masses of partial glycans resulting from fragmentation across glycosidic bonds as well as cross-ring fragments^43^. We found evidence for 21 ions with masses consistent with partial glycans. Interestingly, under the fragmentation conditions used here, partial glycans typically comprised only a portion of the *N*-acetylglucosamine core. The native mass spectrum showed that four proteoforms differ by a consistent mass increase of 162 Da, suggesting the sequential addition of single hexose moieties to the hybrid glycans. In line with the native mass spectrum, the complete composition and occupancy of the *N*-glycans were validated through glycoproteomics (Supplementary Table 2 and Supplementary Fig. 7). Despite the complex fragmentation resulting from the partial loss of hybrid glycans, we successfully achieved substantial sequence coverage of the endogenous receptor.

The proteoform with the highest abundance carries two N-linked glycans at sites Asn2 and Asn15; the glycans at each site are composed of the same combination of three mannose (Man) and three *N*-acetylglucosamine (GlcNAc) residues (Man_3_GlcNAc_3_; Fig. 2a, inset). For the remaining proteoforms, the composition of the N-linked glycan at site Asn2 remains static (Man_3_GlcNAc_3_), while at Asn15 the glycan composition differs by the addition of a single mannose (Man_4_GlcNAc_3_, Man_5_GlcNAc_3_ and Man_6_GlcNAc_3_, respectively). These four proteoforms also contain the two cysteine palmitoylations. Finally, the lowest-molecular-weight proteoform contains two identical hybrid glycans (Man_3_GlcNAc_3_) and only one palmitoylation. All of the proteoforms lacked the retinylidene chromophore, which was expected as the membranes were exposed to ambient light. Altogether, we achieved 14% sequence coverage of an endogenous GPCR. This was sufficient to identify the protein and confirm the presence of several PTMs without having to alter the protein composition through the enzymatic cleavage of glycans^44^ or through chemical derivatization of cysteines^45^.

### Unique proteoforms in the intradiscal cellular space

**Fig. 3:**
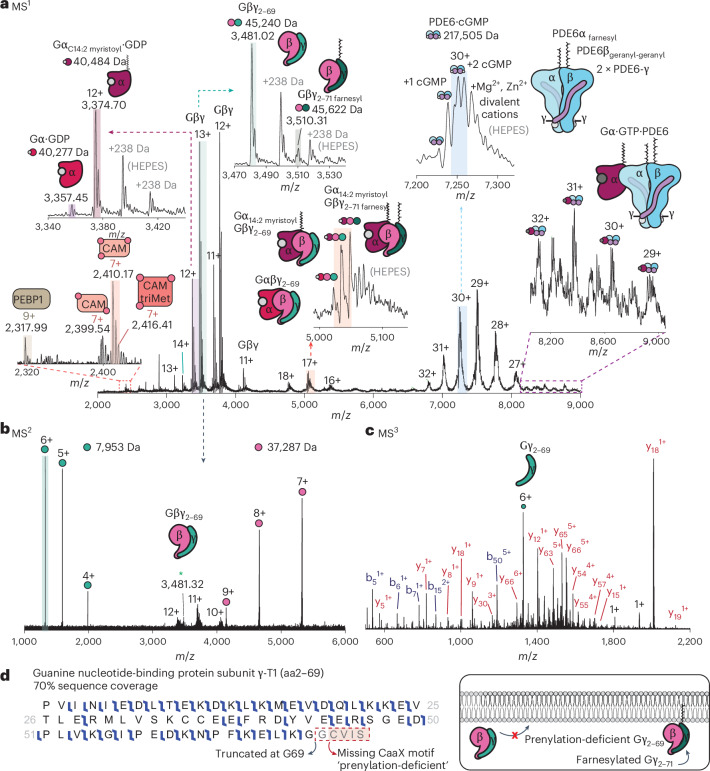
Identification of proteoforms in the intradiscal fraction. **a**, Native mass spectrum of the intradiscal fraction. The highlighted peaks below *m*/*z* = 8,000 were selected and fragmented to confirm the identities of the proteoforms (see Supporting Information for the annotated MS^2^ spectra). triMet, trimethylated. **b**, Quadrupole *m*/*z* filter selection of the Gβγ complex at *m*/*z* = 3,481 by gentle activation (dissociation without fragmentation) successfully dissociated the non-covalently bound Gβ and Gγ subunits. However, the intact mass of the Gγ subunit was lower than the expected theoretical mass. **c**, The 6+ charge state of Gγ at *m*/*z* = 1,391 was subsequently isolated using the ion trap and fragmented in an MS^3^ experiment. **d**, The MS^3^ spectrum confirmed the identity of a truncated form of guanine nucleotide-binding protein subunit γ-T1 (Gγ_1t_) containing amino acids (aa) 2–69 with high confidence at 70% sequence coverage (left). This proteoform lacks the final five residues at the C terminus, comprising the CaaX motif that directs farnesylation to the C terminus, thus making it prenylation-deficient and unable to localize to the membrane (right).

Considering other proteins involved in rhodopsin signalling, we examined the intradiscal fraction of proteins excluded from the vesicles. Many high-molecular-weight distributions above *m*/*z* = 4,500 were observed (Fig. 3a). This indicated that the pure vesicle preparation omitted many membrane-associated proteins expected to be present in higher abundance in the intradiscal environment. We anticipated a variety of dynamic protein–protein and protein–nucleotide complexes; intact masses were first used to infer the protein composition of the complexes, and top-down MS confirmed their identities and PTM composition. Beginning with the lowest *m*/*z* peaks, we identified PEBP1, a known modulator of GPCR signalling^46^ (Supplementary Fig. 8). We also observed a population of CAM bound to two and three Ca^2+^, consistent with predicted models of multi-site binding to cooperatively linked binding centres^47^. Fragments consistent with a trimethylated form of CAM, localized to Lys116, were evident in the MS^2^ spectrum (Supplementary Fig. 9). This proteoform could not be readily distinguished amid the heterogeneous distribution of Ca^2+^-bound states in the MS^1^ spectrum. As no exogenous calcium was added during the preparation of the intradiscal fraction, we have captured the native state calcium occupancy in this signalling environment.

Moving to higher *m*/*z*, we observed a duplet of peaks (12+, *m*/*z* = 3,340–3,430; Fig. 3a, inset), identified as proteoforms of the α subunit of the heterotrimeric guanine nucleotide-binding protein (Gα). The lower abundance distribution (*m*/*z* = 3,357), lacking the initiator methionine, showed no other modifications (Supplementary Fig. 10a,b), but harboured an additional intact mass of 443 Da. This increase corresponds to guanosine diphosphate (GDP), the nucleotide that binds with Gα in its inactive form as a 1:1 complex. The more abundant species was N-terminally modified with a polyunsaturated myristoyl (C14:2), that is, Gα_C14:2 myristoyl_–GDP (Supplementary Fig. 10c,d). Unexpectedly, we did not observe any evidence for the more common C14:0 myristoylation. Given that there is a greater availability of C14:2 and C14:1 N-acylation substrates in the retina compared with C14:0 (ref. ^48^) and that this lipid modification promotes membrane association, the absence of the myristoyl heterogeneity on Gα implies a regulated and specific role of the C14:2 acyl chain^49^.

Turning to the most abundant distribution (13+, *m*/*z* = 3,470–3,540; Fig. 3a, inset), initial fragmentation confirmed the presence of both Gβ and Gγ subunits; two proteoforms of the Gβγ heterodimer were apparent (measured masses of 45,240 and 45,622 Da). No single modification or predictable binding partner could account for the mass difference of 382 Da, prompting further investigation by MS^2^. Isolating the charge state at *m*/*z* = 3,481, we first used low-level infrared activation (~3.6 W) to disrupt the inter-subunit interaction, releasing intact Gβ and Gγ subunits (Fig. 3b). The measured mass of the Gβ subunit is in accord with the mature subunit missing a methionine initiator and harbouring N-terminal acetylation (37,287 Da). Meanwhile, the released Gγ subunit had a lower molecular weight (−382 Da). Isolating its 6+ charge state (*m*/*z* = 1,391) and fragmentation in an MS^3^ experiment (Fig. 3c), we assigned a truncated form of guanine nucleotide-binding protein subunit γ-T1(Gγ_1t_) with high confidence (70% sequence coverage; Fig. 3d). This unique proteoform lacks the final five residues, including the CaaX motif required for directing prenylation to the C terminus. This proteoform, a prenylation-deficient form of Gβγ (Gβγ_2–69_), is unable to localize to the membrane (Fig. 3d). The second higher molecular weight Gβγ proteoform comprised the full-length Gγ (residues 2–71) with a confident assignment of farnesylation at C71 (Supplementary Fig. 11).

In addition to individual G protein subunits, a complex with a heterogeneous mass distribution at *m*/*z* ≈ 5,000 (Fig. 3a) was subjected to MS^2^ analysis. The presence of all three G proteins, Gα, Gβ and Gγ, confirmed the complex as heterotrimeric transducin (Supplementary Fig. 12). The heterogeneity in the peak distribution assigned to transducin suggests the incorporation of different Gα and Gβγ proteoforms. First, the low abundance peak corresponds to a complex of unmodified Gα and prenylation-deficient Gβγ_2__–__69_ (Fig. 3a, inset). This complex is expected to exist entirely in the intradiscal space, being unable to localize to the membrane without lipid modifications. The next complex in this series was assigned to Gα_C14:2 myristoyl_ and prenylation-deficient Gβγ_2__–__69_. Glycine myristoylation alone is often insufficient for membrane localization^50^; this requires a conformational change at the *N*-myristoyl moiety mediated by other proximal lipid modifications (for example, the prenylation of Gγ) or through the binding of the activator guanine triphosphate (GTP), suggesting cytosolic localization of this complex^51^. G protein subunits of transducin have been shown to diffuse into the cytosol and other rod cellular compartments in response to light^52,53^. Turning to the major peak in the distribution, we assign a doubly lipidated trimer comprising Gα_C14:2 myristoyl_ and Gβγ_2__–__71 farnesyl_. Interestingly, despite the low abundance of the free Gβγ_2__–__71 farnesyl_ proteoform, the high abundance of this combination suggests the preferential formation of the doubly lipidated transducin complex.

Finally, the high *m*/*z* distribution (*m*/*z* > 6,500) corresponds to a measured mass of 217,505 Da. Isolation and fragmentation of the 30+ charge state (*m*/*z* = 7,250) yielded sequence ions consistent with subunits of the heterotetrameric enzyme PDE6. PDE6 is an essential effector of visual signal transduction that regulates cyclic guanosine monophosphate (cGMP) levels and thereby regulates cyclic nucleotide-gated channels. PDE6 comprises two different catalytic subunits, PDE6α and PDE6β (ref. ^54^), and two copies of an identical inhibitory gamma subunit, PDE6γ (Supplementary Table 1). The ambiguity in the measured mass, coupled with the overall broadness of the peaks, suggests PTMs to the subunits and/or binding of its substrate, cGMP (Supplementary Table 1). We observed fragment ions consistent with C-terminal farnesylation of PDE6α, while the PDE6β subunit was modified at the C terminus with geranyl-geranylation, a more hydrophobic modification than farnesylation (Supplementary Fig. 13). These modifications are specific to rod PDE6, and both lipid modifications are critical for its function^55^. Two cGMP substrates were bound to the PDE6 complex with additional evidence for binding of a single cGMP and a very low abundant nucleotide-deficient PDE6 (Fig. 3a, inset). Additional adducts were assigned to combinations of metal ions (Zn^2+^ or Mg^2+^) that bind concomitantly with cGMP and help to maintain a low rate of hydrolysis of 3′,5′-cGMP to 5′-GMP (ref. ^56^). Additional adducts were attributed to residual HEPES buffer, which was also evident in other protein distributions. As the catalytic activity of PDE6 is directly regulated by an activated Gα–GTP, we searched for peaks consistent with a Gα–GTP–PDE6 complex. At high *m*/*z* (*m*/*z* > 8,000), we observed a low abundance series of peaks consistent with a 1:1 complex of Gα–GTP with tetrameric PDE6 (Fig. 3a); the low abundance of this complex is likely indicative of its brief lifetime^57^.

**Extended Data Fig. 2:**
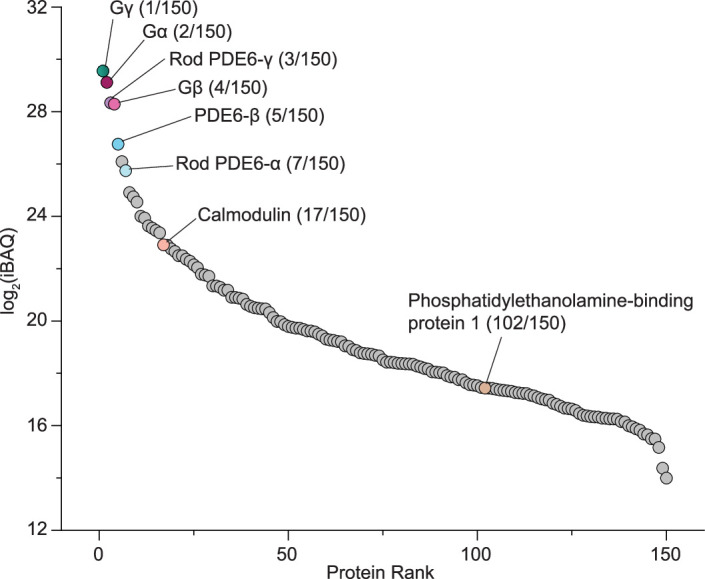
Bottom-up proteomics analysis of rod photoreceptor proteins in the intradiscal space. Plot of each protein ranked by log_2_ of the iBAQ value. The proteins identified by native MS and top-down MS are annotated with their rank order. Source data

As with the rod disc membranes, we surveyed the proteome of the intradiscal fraction using bottom-up proteomics (Extended Data Fig. 2). Again, we observed a disparity in the proteins observed in the native mass spectrum and their relative abundances determined by proteomics. Similarly, PEBP1 was ranked 102 out of 150 proteins detected by bottom-up MS. However, we readily observed this protein by native MS with adequate intensity for proteoform-level characterization by top-down MS (Supplementary Fig. 8). This lipid-binding protein undertakes its functional role in proximity to membranes, further suggesting that the proteins observed in the native mass spectrum are representative of their organization within the native environment.

### Off-target drug binding at the single proteoform level

Our confident assignment of individual proteoforms in the membrane and intradiscal cellular space presented a unique opportunity to directly evaluate drug binding to proteins within their endogenous environment. We selected two PDE inhibitors, vardenafil and sildenafil, whose intended target is PDE5 found in the membranes of blood vessels. These drugs possess the therapeutic ability to relax blood vessels through the inhibition of cGMP hydrolysis and are most widely used to treat erectile dysfunction^58^. However, both drugs are also capable of crossing the blood–retina barrier and are known to cross-react with retina PDE6 (ref. ^59^). As a consequence, these inhibitors have been linked to undesirable ocular side-effects which typically manifest as temporary changes to light and colour perception, and occasionally as more severe problems such as damage to the optic vasculature^60^.

**Fig. 4:**
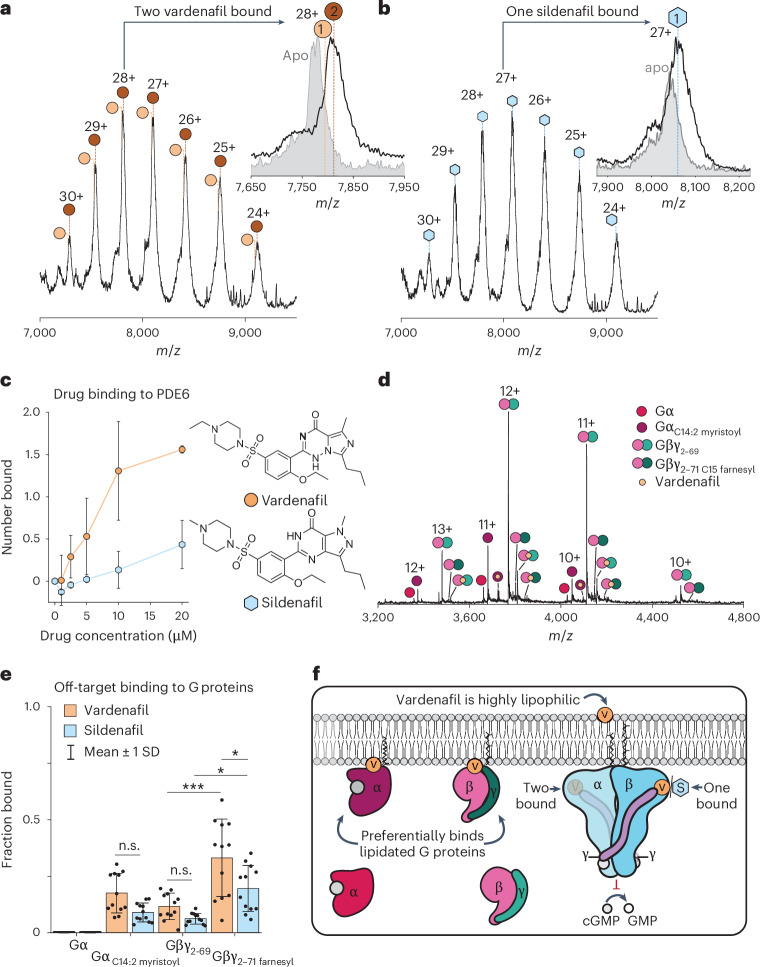
Off-target drug binding to retina proteins in the intradiscal space. **a**,**b**, Mass spectra of PDE6 incubated with 20 µM vardenafil (**a**) and 20 µM sildenafil (**b**). Insets: magnifications of the main charge state with the drug bound (white peak) and the equivalent charge state without drug binding (grey peak). **c**, Titration of vardenafil (orange circles) and sildenafil (blue hexagons) to evaluate drug binding to PDE6, represented as the number of drugs bound versus drug concentration. The error bars represent ±1 standard deviation for *n* = 6 technical replicates. **d**, Evidence for additional off-target drug binding to G proteins in the intradiscal fraction incubated with 20 µM vardenafil. **e**, Bar chart showing the amount of off-target drug binding to G protein proteoforms represented as the fraction bound. The heights of the bars denote the mean and the error bars represent ±1 standard deviation from the mean. Statistical significance was established using one-way analysis of variance (ANOVA) with a post hoc Bonferroni correction for multiple comparisons for *n* = 12 technical replicates. Statistically significant differences were observed for vardenafil binding to Gβ–Gγ_2__–__69_ versus Gβ–Gγ_2__–__71 farnesyl_ (***P < 0.0001), sildenafil binding to Gβ–Gγ_2__–__69_ versus Gβ–Gγ_2__–__71 farnesyl_ (*P = 0.013) and vardenafil versus sildenafil binding to Gβ–Gγ_2__–__71 farnesyl_ (*P = 0.0116). n.s., not significant. **f**, Schematic showing the proposed mechanism of binding, where lipophilic vardenafil can permeate the membrane and preferentially interact with the hydrophobic lipid modifications on the G proteins and PDE6, facilitating drug transfer to the catalytic sites of PDE6α and PDE6β, where both sildenafil and vardenafil can bind. Source data

To probe their interaction with rod photoreceptor PDE6 in the context of its native environment, we introduced each drug directly into the intradiscal fraction. In the presence of vardenafil, the mass distribution of PDE6 increased by 976 Da, corresponding to the binding of two vardenafil molecules (Fig. 4a). Interestingly, the same concentration of sildenafil increased the mass of PDE6 by only 476 Da, the mass of a single molecule of sildenafil (Fig. 4b). Vardenafil has been found to be ten times more potent as an inhibitor of PDE6 than sildenafil^61^. To investigate the apparent differences in binding, we titrated both drugs to determine the fraction of drug bound to PDE6 relative to the concentration of the drug (Fig. 4c). At the lower drug concentrations tested, PDE6 readily bound two vardenafil molecules. In contrast, PDE6 never bound more than one sildenafil molecule at any of the concentrations tested (Fig. 4c).

To determine whether additional off-target interactions occurred with other proteins, we looked for evidence of new peaks in the mass spectra that would indicate drug binding. To our surprise, we found evidence for vardenafil binding to G protein subunits (Fig. 4d). Sildenafil also exhibited off-target binding to G proteins, however, to a much lesser extent (Fig. 4e and Supplementary Figs. 14 and 15). Notably, we found a statistically significant increase in binding to lipid-modified G protein subunits compared with their unmodified counterparts for both drugs (Fig. 4e). Of note, vardenafil is highly lipophilic and is considered a high-permeability, low-solubility drug^62^. It is interesting to note that the farnesyl and geranyl-geranyl modifications on PDE6α and PDE6β are proximal to the sites at which both drugs bind. These modifications create a possible conduit for vardenafil to interact hydrophobically with the protein before binding to the catalytic sites of PDE6α and PDE6β. This drug binding to membrane-associated proteoforms implies that hydrophobic modifications enhance off-target binding (Fig. 4f), potentially influencing the occurrence of unwanted side-effects as well as the severity of their presentation.

## Discussion

We have presented herein a technological advance in native top-down MS, where an infrared laser is directed into a linear ion trap of a mass spectrometer to release a GPCR and its effectors directly from a native lipid bilayer. In addition, we have demonstrated the controlled stepwise release of proteins from the bilayer, followed by their top-down sequencing via IRMPD. For rhodopsin, we observed five distinct proteoforms with differences in glycosylation and palmitoylation. In addition, we identified over a dozen retina proteins directly involved in the phototransduction cascade. The corresponding fragmentation spectra were densely packed with information, allowing us to define N- and C-terminal truncations as well as a complex PTM status. We further identified a plethora of lipid modifications to G proteins and PDE6, including farnesylation, geranyl-geranylation and an unusual polyunsaturated myristoylation as well as a prenylation-deficient form of the heterodimeric Gβγ complex.

**Fig. 5:**
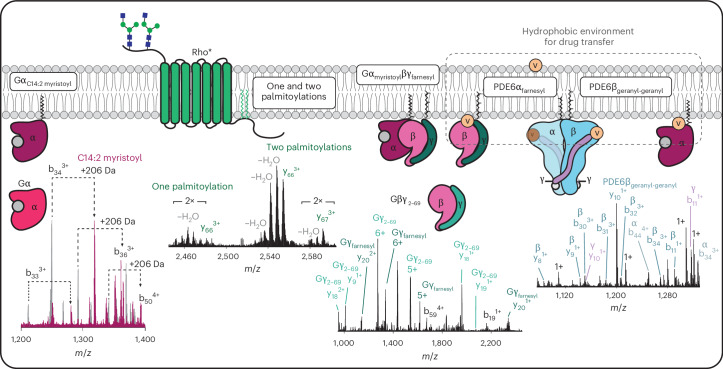
Summary of lipid modifications detected in proteins in rod photoreceptor membranes. The fragmentation spectrum of Gα reveals an uncommon polyunsaturated myristoylation (C14:2). Rhodopsin was found to harbour one and two palmitoyl moieties at two cysteines near the C terminus. Two forms of Gβγ heterodimers have been identified: one prenylation-deficient form of Gγ and another form containing farnesylation on C71. Finally, two different lipid modifications have been detected in subunits of PDE6: farnesylation of the α subunit and geranyl-geranylation of the β subunit. Doubly lipidated PDE6 interacts with vardenafil, a PDE5 inhibitor, binding two molecules, one at each catalytic site of the heterodimer. Vardenafil also binds preferentially to farnesylated Gβγ and myristoylated Gα. We propose that the hydrophobic environment created by lipid modifications creates a conduit for lipophilic drug transfer.

Of particular note is the retention of lipid modifications, especially palmitoylation, amidst extensive backbone fragmentation. Palmitoylation is often difficult to observe due to a tendency to break from the protein during MS/MS analysis^41^. This level of molecular detail is important in understanding disease pathogenesis. For example, our data reveal complete lipidation of both PDE6α and PDE6β subunits. Defects in the lipidation of PDE6 subunits in particular have been linked to the degeneration of rods leading to blindness^63^. Furthermore, we discovered a prenylation-deficient Gγ proteoform that can still interact with Gα. A study of the role of Gγ farnesylation in rod photoreceptors of mice similarly found that prenylation-deficient Gβγ dimers could participate in heterotrimeric G protein formation, however, the complex could not mediate phototransduction^64^. As Gβγ heterodimers have been found to participate in receptor-independent signalling pathways in other subcellular compartments^65^, our observation of its presence lends further support to its additional roles beyond classical GPCR signalling (Fig. 5).

Finally, given the important role of hydrophobic lipid modifications in defining cellular localization and interactions with signalling proteins, this particular aspect of our experimental platform is of increasing importance for understanding subtle molecular changes that can influence drug discovery^66^. Our detailed characterization of proteoforms enabled us to study drug binding within the cellular environment, revealing the effects of two PDE5 inhibitors and their cross-reactivity with lipidated proteins. We have provided direct evidence for differential drug binding to PDE6, where vardenafil binds both the PDE6α and PDE6β catalytic domains, an effect that was not observed with sildenafil. This result is important considering that rod photoreceptor PDE6 is uniquely a heterodimer comprising different α and β subunits with farnesylation and geranyl-geranylation, respectively. In contrast, other type I PDEs, including cone photoreceptor PDE6, comprise two identical α subunits and, importantly, do not harbour hydrophobic modifications.

We have also revealed differential drug interactions with lipidated G proteins. The higher drug-binding occupancy of vardenafil compared with sildenafil can be explained by its increased hydrophobicity. As lipidations are known to increase interactions with the membrane bilayer and mediate hydrophobic interactions, the likely segregation of these lipidated proteins provides a hydrophobic microenvironment that could facilitate the transfer of drugs. We note that off-target effects of seemingly unrelated medications in the context of protein prenylation have been reported previously^66^ and speculate that such mechanisms could also be operative here.

In light of these developments in native top-down MS, we consider the current limitations and future directions. Native top-down MS is challenged by a low overall sequence coverage of high-molecular-weight proteins. Technological innovations^67–70^, including the infrared-enabled mass spectrometer described here, have largely overcome the challenges associated with generating rich fragmentation spectra from intact proteins. Emerging now are challenges for informatics to unambiguously identify PTMs and amino acid variations amid the vast and heterogeneous proteoform landscape. In fact, even for relatively simple systems, many fragments are left unassigned due to the computational burden of searching all possible proteoforms and making accurate assignments amid false discoveries. Moreover, there are expected to be many unannotated PTMs, necessitating improvements in search strategies to expand beyond known proteoforms. We envision that new data analysis approaches will enhance our ability to discover proteoforms, accelerating biological discoveries linking PTMs with interactions through native top-down MS.

In summary, we have offered a detailed molecular snapshot of the dynamic and heterogeneous cellular environment found within retina membranes and in the intradiscal fraction. The capacity to sequence proteins and their proteoforms in this native context has enabled us to assess individual drug-binding events at the single proteoform level directly within this endogenous environment. Although our current focus lies on the well-studied rhodopsin signalling system, we foresee that our ability to perform top-down sequencing from native membranes and to define multiple lipidated states and proteoforms will significantly enhance our capacity for drug discovery in increasingly complex native tissue environments.

## Methods

### Preparation of rod disc membranes and intradiscal fraction

Rod disc membranes and intradiscal fractions were kind gifts from K. Palczewski (University of California, Irvine) and were prepared from commercially obtained bovine retinas as described previously^31^. Briefly, rod disc membranes were isolated from a batch of 50–100 retinas using stepped sucrose gradient ultracentrifugation^71–73^. Soluble and membrane-associated proteins, which typically co-migrate with the membranes, were partly removed by washing with isotonic buffer comprising 20 mM 4-(2-hydroxyethyl)-1-piperazineethanesulfonic acid (HEPES), pH 7.5, 100 mM NaCl, 1 mM dithiothreitol (DTT) and 5 mM MgCl_2_, followed by centrifugation at 31,000 *g* (25 min, 4 °C). This fraction was retained and is referred to herein as the excluded intradiscal fraction. The washed membranes were disrupted via sonication to form vesicles using a modified protocol similar to that previously described^74^. Isolated membranes were then suspended in 200 mM ammonium acetate and homogenized using a probe sonicator with a stepped tip microtip (2 mm, Vibra-Cell VCX-500, Sonics) and a maximal amplitude (40%, 1 s on, 2 s off), applying 2 J per cycle for 1.5 min (ref. ^31^). The vesicles were concentrated using a 10 kDa molecular weight cut-off (MWCO) centrifugal concentrator (Amicon) until the concentration of total protein in the vesicles containing rhodopsin was ~9 µM (~0.4 mg ml^−1^), as determined by UV absorbance at 280 nm. The disc membrane vesicles were diluted twofold with 200 mM ammonium acetate and the excluded intradiscal fraction was buffer-exchanged into 200 mM ammonium acetate using a desalting column (Zeba spin, 7,000 MWCO). Approximately 3 µl was loaded into a gold-coated glass capillary for native MS analysis.

### Glycoproteomics

Rhodopsin was first purified from rod disc membranes and solubilized in detergent before glycoproteomic analysis. Briefly, membranes were solubilized in 1% lauryl maltose neopentyl glycol (LMNG) with 0.1% cholesterol hemisuccinate (CHS) overnight at 4 °C. The solubilized lysate was clarified by centrifugation at 20,000 *g* and the supernatant was loaded onto 1D4 magnetic agarose beads (Cube Biotech). After incubation for 2–3 h at 4 °C, the beads were washed ten times with ten times the bead volume of 20 mM HEPES, pH 8, and 200 mM NaCl supplemented with twice the critical micelle concentration of LMNG (0.002%, w/v) supplemented with 0.01% CHS. Rhodopsin was eluted in 500 µl via competitive elution with 1 mg ml^−1^ 1D4 peptide suspended in the same wash buffer as above. Excess 1D4 elution peptide was removed while simultaneously concentrating the purified rhodopsin using a 100 kDa MWCO centrifugal filter (Amicon).

Approximately 5 µg rhodopsin was reduced, loaded and run on SDS–PAGE. The gel bands were excised and washed sequentially with HPLC-grade water and 1:1 (v/v) MeCN–H_2_O. The gel bands were then dried (via vacuum centrifugation), treated with 10 mM DTT in 100 mM NH_4_HCO_3_ and incubated for 45 min at 56 °C with shaking. DTT was then removed and 55 mM iodoacetamide (in 100 mM NH_4_HCO_3_) was added and incubated for 30 min in the dark. All of the liquid was removed and the gels were washed with 100 mM NH_4_HCO_3_–MeCN as above. The gels were dried, rehydrated separately with 12.5 ng µl^−1^ chymotrypsin and incubated overnight at 37 °C. The rehydrated gels were then washed and peptides were extracted and pooled with sequential washes with 5% (v/v) formic acid in H_2_O and MeCN. The dried peptides were reconstituted in 2% MeCN–0.05% trifluoroacetic acid and analysed by LC–MS.

The peptides were analysed with three technical replicates using an Ultimate 3000 UHPLC device coupled to an Orbitrap Q Exactive mass spectrometer (Thermo Fisher Scientific). The peptides were loaded onto a 75 µm × 2 cm pre-column and separated on a 75 µm × 15 cm Pepmap C18 analytical column (Thermo Fisher Scientific). Two buffers were prepared; buffer A comprised 0.1% formic acid in H_2_O and buffer B comprised 0.1% formic acid in 80% MeCN with 20% H_2_O. A 100-min linear gradient (0–40% buffer B) was used. A universal higher-energy collisional dissociation (HCD) identification method was used. Data were collected in data-dependent acquisition mode with an *m*/*z* range of 375–1,500 at a resolution of 70,000. For MS/MS analysis, the stepped HCD normalized energy was set to 27, 30 and 33 with Orbitrap detection at a resolution of 35,000 at *m*/*z* = 200.

The glycopeptide data were analysed using the Byonic (Protein Metrics, https://proteinmetrics.com/byonic/) software (version 5.4.10). Digestion was set to FYWML for chymotrypsin and fully specific with a maximum of two missed cleavages allowed. Carbamidomethylation (57.02 Da) was set as a fixed modification, while methionine oxidation (15.99 Da), deamidation (0.98 Da) and glutamine to pyroglutamate (−17.03 Da) were set as variable modifications. The Byonic in-built common human N-linked (132 glycans) and O-linked glycan (9 glycans) databases were used to identify glycopeptides. Byonic output files were imported into Byologic (Protein Metrics) for quantification. All glycopeptide assignments were manually validated.

### Bottom-up LC–MS

Proteins (∼10–50 μg) were denatured in 4.5 M urea (in 100 mM ammonium bicarbonate, pH 7.5, Sigma-Aldrich). Disulfide bonds were reduced in 1 mM tris(2-carboxyethyl)phosphine hydrochloride (Sigma-Aldrich) at 56 °C for 0.5 h. The reduced cysteines were subsequently alkylated with iodoacetamide (1 mM, Sigma-Aldrich) in the dark (1 h, room temperature). The urea was diluted to 1 M with 100 mM ammonium bicarbonate, followed by the addition of trypsin (1:50 (w/w), modified sequencing-grade trypsin, Promega). Proteins were allowed to digest overnight at 37 °C. Peptides were desalted using Pierce C18 spin tips (100 µl) and then dried in a vacuum concentrator. The desalted peptides were resolubilized in buffer A (0.1% formic acid, LC–MS-grade H_2_O, Fisher Scientific) and loaded onto a reversed-phase trap column (Acclaim PepMap 100, 75 μm × 2 cm, nanoviper, C18, 3 μm, 100 Å, Thermo Fisher Scientific) using an Ultimate 3000 device (Thermo Fisher Scientific) at a flow rate of 20 μl min^−1^. The trapped peptides were washed with 60 µl of buffer A and then analytically separated (Acclaim PepMap RSLC, 75 μm × 50 cm, 2 μm, 100 Å, ThermoFisher) using a 100 min linear gradient of 5–40% buffer B (0.1% formic acid, 80% acetonitrile, 20% H_2_O, Fisher Scientific) at a flow rate of 300 nl min^−1^. The separated peptides were then electrosprayed in positive mode into an Orbitrap Eclipse Tribrid mass spectrometer operated in data-dependent mode (3 s cycle time). Precursor peptides were analysed in the Orbitrap analyser (resolution of 120,000 at *m*/*z* =200 , *m*/*z* range of 300–2,000, 100% automatic gain control (AGC)). Precursor peaks above an intensity threshold of 5.0 × 10^4^, corresponding to multiply charged peptide ions (charge states 2–5), were selected with the quadrupole using a 0.7 *m*/*z* selection window and fragmented using HCD (30% normalized energy). MS^2^ spectra of dissociated peptide ions were collected in the Orbitrap (resolution of 30,000 at *m*/*z* = 200, 100% AGC). Subsequent MS^2^ scans of like precursor ions within 10 ppm were dynamically excluded after the initial scan for 30 s. Data were collected in triplicate (*n* = 3 independent digestions).

### LC–MS data analysis

LC-MS data were searched against the bovine proteome (downloaded from Swiss Prot on 7 August 2024) using Maxquant (version 2.3.1.0, https://www.maxquant.org/) with default settings. Label-free quantification (LFQ) and intensity-based absolute quantification (iBAQ) were enabled (*n* ≥ 2 peptides for LFQ). Oxidation of methionine and N-terminal acetylation were set as variable modifications, and cysteine carbamidomethylation was set as a static modification. The ‘match between runs’ option was enabled. Perseus (version 2.0.11.0, https://www.maxquant.org/perseus/) was used for subsequent analyses. Matched protein groups corresponding to contaminants, those identified with only modified peptides and those that matched the reverse database were removed. iBAQ values were log_2_-transformed before trimming the matched protein groups to only those identified in fewer than two of the three technical replicates. Missing values were imputed using the default settings. Mean iBAQ values were calculated (*n* = 3 technical replicates) and plotted against the ranked order of the identified proteins, sorted by abundance.

### Native MS and top-down MS

Each mass spectrum represents a technical replicate with a high degree of biological heterogeneity (50–100 eyes). Experiments were conducted on an Orbitrap Ascend Tribrid mass spectrometer with two key modifications: (1) a quadrupole mass filter that enabled *m*/*z* selection up to *m*/*z* = 8,000 and (2) addition of a 10.6 µm wavelength CO_2_ laser (Synrad Firestar Ti60 CO_2_ continuous-wave infrared laser) directed into the high-pressure cell of a linear ion trap analyser. The timing and power output were controlled using modified instrument software. The same laser modification to a similar mass spectrometer (Orbitrap Eclipse) has been described previously^26^. The instrument was operated in high-pressure mode (20 mtorr in front and back ion-routing multipoles (IRMs)). Typical source parameters were 900–1,100 V electrospray voltage, 150 °C transfer tube temperature, Orbitrap resolution of 15,000, maximum injection time of 100 ms, 150% radio frequency (RF) lens, 50 V source collision-induced dissociation (CID) and a source CID compensation scaling factor of 0.01. Source CID compensation is a feature available in Tribrid mass spectrometers that serves to better desolvate native proteins by removing adducts and improve the transmission of proteins and complexes through kinetic energy management^75^. The value represents a percentage (ranging from 0.01 to 1), which sets a slowing potential; multipole (MP) MP0 is raised above MP00 by a percentage relative to the source CID voltage. In our experiments, the source CID was set to 50 V and a minimum compensation value of 0.01 was used, corresponding to a slowing potential of 0.5 V.

Vesicles and intradiscal fractions were ionized via nanoelectrospray in gold-coated borosilicate glass capillaries (1.2 mm outer diameter) prepared in house. The capillary voltage was held at 1.0–1.2 kV relative to the instrument orifice (heated at ∼100–200 °C). Ions were activated in the source using a gentle activation energy (50 V source CID) before entering the next differential pressure region containing a curved quadrupole ion guide. The instrument was operated in high-pressure mode (20 mtorr in both ion routing multipoles) and ions were irradiated in the ion trap for 5–25 ms at laser output powers in the range of 0–6 W before being transferred back into the Orbitrap for MS^1^ detection. MS^1^ spectra were collected at a resolution of 15,000 at *m*/*z* = 200. For the MS^2^ experiments, ions were isolated in the ion trap (25–35 *m*/*z* isolation window with a *Q* value of 0.1) and irradiated with the infrared laser for 5 or 10 ms at laser output powers in the range of 6.0–12.0 W to induce fragmentation. For fine precursor selection within the intradiscal fractions, ions were selected in the quadrupole using a 5 *m*/*z* window. Ions were detected in the Orbitrap at a resolution of 240,000 at *m*/*z* = 200. For the MS^3^ experiments, ions were isolated in the ion trap (5–10 *m*/*z* isolation window, *Q* value of 0.1) and subjected to HCD (70–80 V absolute), followed by *m*/*z* analysis in the Orbitrap at a resolution of 240,000 at *m*/*z* = 200.

### Drug binding

Sildenafil citrate and vardenafil HCl trihydrate (ApexBio) were dissolved in dimethylsulfoxide (DMSO) to a concentration of 2 mg ml^−1^ and further diluted in 200 mM ammonium acetate solution such that the final concentration of DMSO was <1%. The intradiscal fraction containing PDE6 was buffer-exchanged once into 200 mM ammonium acetate using a desalting column (Zeba spin, 7,000 MWCO). Then, 1 µl of the intradiscal fraction (0.3 µg µl^−1^) was incubated with 1 µl of drug (5, 12.5, 25, 50, 100 and 250 µM) and further diluted with 3 µl ammonium acetate, yielding solutions containing 300 ng protein with drug concentrations of 0, 1, 2.5, 5, 10, 20 and 50 µM. Controls contained no drug but were diluted with the equivalent DMSO-containing buffer. The intradiscal fraction was allowed to incubate with the drug at 4 °C for 30 min before analysis. Drug-binding measurements were performed with *n* > 6 technical replicates for each drug concentration, where the drug was titrated into a new aliquot of the intradiscal fraction and a new electrospray capillary was prepared and loaded for each measurement to capture the variability due to the emitter and spray conditions. The fraction of drug bound was determined by integrating the area under the peak for the drug-bound charge state and normalizing the value to the total area of the peaks for the apoproteoforms using OriginPro 2024b (version 10.1.5.132).

### Data analysis and software

Proteins were first identified through a database search of the entire bovine proteome using Prosight Native (Proteinaceous)^76^. MS^2^ spectra were searched using the following settings: intact mass search on, fragmentation search on, delta M mode off, subsequence search off, precursor target tolerance of 0.25 *m*/*z*, precursor tolerance of 100–1,000 Da (depending on the mass uncertainty from the precursor protein distribution), fragmentation tolerance of 20.0 ppm, a maximum number of five PTMs per isoform, a maximum number of five PTMs per proteoform, a maximum number of one single nucleotide polymorphism (SNP) per isoform, maximum mass to search of 70,000 Da for all proteins except PDE6, which was set to 100,000 Da, and a thorough high resolution analysis of spectra by Horn (THRASH) window size of 1. Fragments were then manually validated in TDValidator (Proteinaceous)^77^. Spectra were first annotated using a signal-to-noise cut-off of 7, a maximum ppm tolerance of 10 ppm, a sub-ppm tolerance of 5 ppm, a cluster tolerance of 0.35 and a minimum score of 0.6 using the distribution generator Mercury7. Assigned peaks were then manually validated and incorrect assignments were removed. The statistical significance of drug binding to G protein proteoforms was established by one-way ANOVA with a post hoc Bonferroni correction for multiple comparisons using OriginPro 2024b (version 10.1.5.132, https://www.originlab.com/origin).

### Reporting summary

Further information on research design is available in the Nature Portfolio Reporting Summary linked to this article.

## Online content

Any methods, additional references, Nature Portfolio reporting summaries, source data, extended data, supplementary information, acknowledgements, peer review information; details of author contributions and competing interests; and statements of data and code availability are available at 10.1038/s41557-024-01711-w.

## Supplementary information

Supplementary Information Supplementary Tables 1 and 2 and Figs. 1–15.

Reporting Summary

## Source data

Source Data Fig. 4 Statistical source data for Fig. 4c,e.

Source Data Extended Data Fig. 1 Statistical source data for Extended Data Fig. 1.

Source Data Extended Data Fig. 2 Statistical source data for Extended Data Fig. 2.

## Data Availability

The data supporting the main conclusions of this study are presented in the Article and its Supplementary Information. Raw mass spectrometry data that support figures 1–5 are available via Figshare at 10.6084/m9.figshare.25782309 (ref. ^78^). The mass spectrometry proteomics data in Extended Data Figs. 1 and 2 have been deposited at the ProteomeXchange Consortium via the PRIDE^79^ partner repository with the dataset identifier PXD057262. Source data are provided with this paper.
