# Supplementary material for: Defining proteoform-specific interactions for drug targeting in a native cell signalling environment
Source: Nat Chem. 2025 Jan 13;17(2):204–14. doi: 10.1038/s41557-024-01711-w (PMC11794133; doi:10.1038/s41557-024-01711-w)
Supplement: Supplementary file 1 — Supplementary Tables 1 and 2 and Figs. 1–15. [file 41557_2024_1711_MOESM1_ESM.pdf]

nature chemistry

Supplementary information https://doi.org/10.1038/s41557-024-01711-w

Defining proteoform-specific interactions for drug targeting in a native cell signalling environment

In the format provided by the authors and unedited

Table of Contents

Table S1. List of proteoforms identified by native MS and native top-down MS 2

Figure S1. Effect of laser output power on the liberation of membrane proteins from the bilayer. 5

Figure S2. Identification of phosphoglycerate kinase 1 (PGK1). 6

Figure S3. Identification of alpha enolase (ENOA). 7

Figure S4. Identification of two forms of ATP synthase subunit beta 8

Figure S5. Identification of malate dehydrogenase 9

Figure S6. Identification of S-arrestin 10

Table S2. Glycopeptides detected by glycoproteomics 11

Figure S7. N-glycan composition of rhodopsin 12

Figure S8. Identification of phosphatidylethanolamine-binding protein 1 13

Figure S9. Identification of calmodulin 14

Figure S10. Identification of two proteoforms of Gα. 15

Figure S11. Identification of two proteoforms of GβGγ heterodimers. 16

Figure S12. Identification of G protein subunits of the transducin complex. 17

Figure S13. Identification of phosphodiesterase 6 (PDE6). 18

Figure S14. Sildenafil binds to G proteins. 19

Figure S15. Release of drugs bound to Gβγ heterodimers 20

Table S1. List of proteoforms identified by native MS and native top-down MS. Protein ID, modifications detected by MS^2, theoretical average mass, and measured average mass and uncertainty. The measured masses represent the average mass of the charge state distribution using the apex of each peak. The uncertainty reported is calculated as the standard deviation of the mass using at least three different charge states.

**Protein ID**	**Modifications**	**Theoretical average mass (Da)**	**Measured average mass (Da) and uncertainty**
Phosphoglycerate kinase 1	• Loss of initiator Met<br>• acetylation at S2	44,448.44	44,449.15 ± 0.34
Alpha enolase	• loss of initiator Met<br>• Acetylation at S2	47,236.98	47,194.28 ± 0.86*
ATP Synthase subunit beta, Proteofrom 1	• Loss of transit peptide (aa1-46)	51,705.13	51,704.02 ± 0.36
ATP synthase subunit beta, proteoform 2	• Loss of transit peptide (aa1-46)<br>• additional loss of Alanine (aa47)	51,634.05	51,634.04 ± 0.45
Creatine kinase (dimer)	• Loss of initiator methionine	85,176.40	85,181.63 ± 0.51
Malate dehydrogenase (mitochondrial, dimer)	• Loss of transit peptide (aa1-24)	66,391.38	66,390.79 ± 0.04
Visual arrestin (S-arrestin)	• N-terminal acetylation	45,316.93	45,315.62 ± 0.44
Phosphatidylethanolamine binding protein 1	• Loss of initiator methionine	20,854.55	20,854.08 ± 0.48
Calmodulin	• Loss of the initiator methionine<br>• Acetylation at serine 2<br>• Bound to 3 Ca^{2+} (small population bound to 2 Ca^{2+})<br>• Evidence for trimethyllysine at K116 in MS^2 spectrum	16,748.42 (apo)<br>16,788 (1x Ca^{2+})<br>16,828.58 (2x Ca^{2+})<br>**16,868.66 (3x Ca^{2+})**<br>16,908.74 (4x Ca^{2+})	16,865.33 ± 0.30
Guanine nucleotide binding protein α proteoform 1 (Gα·GDP)	• Loss of initiator methionine<br>• Bound to GDP	39,834.49 (apo)<br>**40,277.69 (+GDP)**	40,277.53 ± 0.14
Guanine nucleotide binding protein α proteoform 2 ($G\alpha_{C14:2\ myristoyl}$·GDP)	• Loss of initiator methionine<br>• C14:2 myristoylation at Glycine2<br>• Bound to GDP	40,040.65 (apo)<br>**40,483.85 (+GDP)**	40,484.26 ± 0.07
Guanine nucleotide binding protein β (Gβ)	• Loss of initiator methionine<br>• Acetylation at serine 2	37,287.80	37,287.52 ± 0.15

Guanine nucleotide binding protein γ proteoform 1 (Gβ·Gγ$_{2-69}$)	• Loss of initiator methionine<br>• Loss of propeptide VIS at C-terminus<br>• Additional loss of GC at C-terminus	7,953.15	7952.06 ± 0.01
Guanine nucleotide binding protein γ proteoform 2 (Gβ·Gγ$_{2-71\ farnesyl}$)	• Loss of initiator methionine<br>• Loss of propeptide VIS at C-terminus<br>• Farnesylation at cysteine 71	8,317.52	8316.31 ± 0.00
PDE6α	• Loss of initiator Met<br>• loss of propeptide CVQ at c-terminus<br>• N-acetylglycine<br>• Farnesylation at C856	99,125.75	Not individually measured (see below for complex mass)
PDE6β	• Loss of initiator met<br>• loss of propeptide RIL at c-terminus<br>• N-acetylserine:<br>• Geranyl geranylation at C850	98,131.58	Not individually measured (see below for complex mass)
PDE6γ	• N-terminal acetylation	9,711.26	Not individually measured (see below for complex mass)
Rhodopsin (proteoform 1)	• N-terminal acetylation<br>• $Man_3GlcNAc_3$ at Asparagine 2<br>• $Man_3GlcNAc_3$ at Asparagine 15<br>• Disulfide bond C110-C187<br>• 1x Palmitoylation at C322 or 323	41,476.03	41,479.29 ± 1.79
Rhodopsin (proteoform 2)	• N-terminal acetylation<br>• $Man_3GlcNAc_3$ at Asparagine 2<br>• $Man_3GlcNAc_3$ at Asparagine 15<br>• Disulfide bond C110-C187<br>• 2x Palmitoylation at C322 and 323	41,714.43	41,716.85 ± 0.83
Rhodopsin (proteoform 3)	• N-terminal acetylation<br>• $Man_3GlcNAc_3$ at Asparagine 2<br>• $Man_3GlcNAc_4$ at Asparagine 15	41,876.58	41,879.90 ± 0.40

	• Disulfide bond C110-C187<br>• 2x Palmitoylation at C322 and 323		
Rhodopsin (proteoform 4)	• N-terminal acetylation<br>• $Man_3GlcNAc_3$ at Asparagine 2<br>• $Man_3GlcNAc_5$ at Asparagine 15<br>• Disulfide bond C110-C187<br>• 2x Palmitoylation at C322 and 323	42,038.70	42,041.08 ± 0.65
Rhodopsin (proteoform 5)	• N-terminal acetylation<br>• $Man_3GlcNAc_3$ at Asparagine 2<br>• $Man_3GlcNAc_6$ at Asparagine 15<br>• Disulfide bond C110-C187<br>• 2x Palmitoylation at C322 and 323	42,200.86	42,203.12 ± 0.65

Masses of protein complexes**

**Complex**	**Theoretical mass (Da)**	**Measured mass (Da)**
Gβ·Gγ$_{2\text{-}69}$	45,240.95	45,239.95 ± 0.48
Gβ·Gγ$_{2\text{-}71\ \text{farnesyl}}$	45,605.32	45,621.52 ± 0.83
Gα·Gβ·Gγ$_{2\text{-}69}$	85,518.64	85,527.95 ± 1.52
Gα$_{\text{C14:2 myristoyl}}$·Gβ·Gγ$_{2\text{-}69}$	85,724.80	85,728.39 ± 0.15
Gα$_{\text{C14:2 myristoyl}}$·Gβ·Gγ$_{2\text{-}71\ \text{farnesyl}}$	86,089.17	86,113.93 ± 1.38
PDE6α$_{\text{farnesyl}}$·PDE6β$_{\text{geranyl-geranyl}}$·2x PDE6γ· 2x cGMP	217,370.27	217,505 ± 30
Gα$_{\text{C14:2 myristoyl}}$·GTP· PDE6α$_{\text{farnesyl}}$·PDE6β$_{\text{geranyl-geranyl}}$·2x PDE6γ· 2x cGMP	257,934.1	263,051 ± 64

*see Figure S3 for a discussion on the mass discrepancy of -42 Da

** The mass discrepancy of protein complexes tends to bias toward positive error (i.e., the measured mass is higher than the theoretical mass) which can be attributed to adduction of salts and cofactors, as well as the presence of multiple coexisting stoichiometries of proteoforms. These effects will shift the apex of each charge state and can result in higher uncertainties in the measured mass.

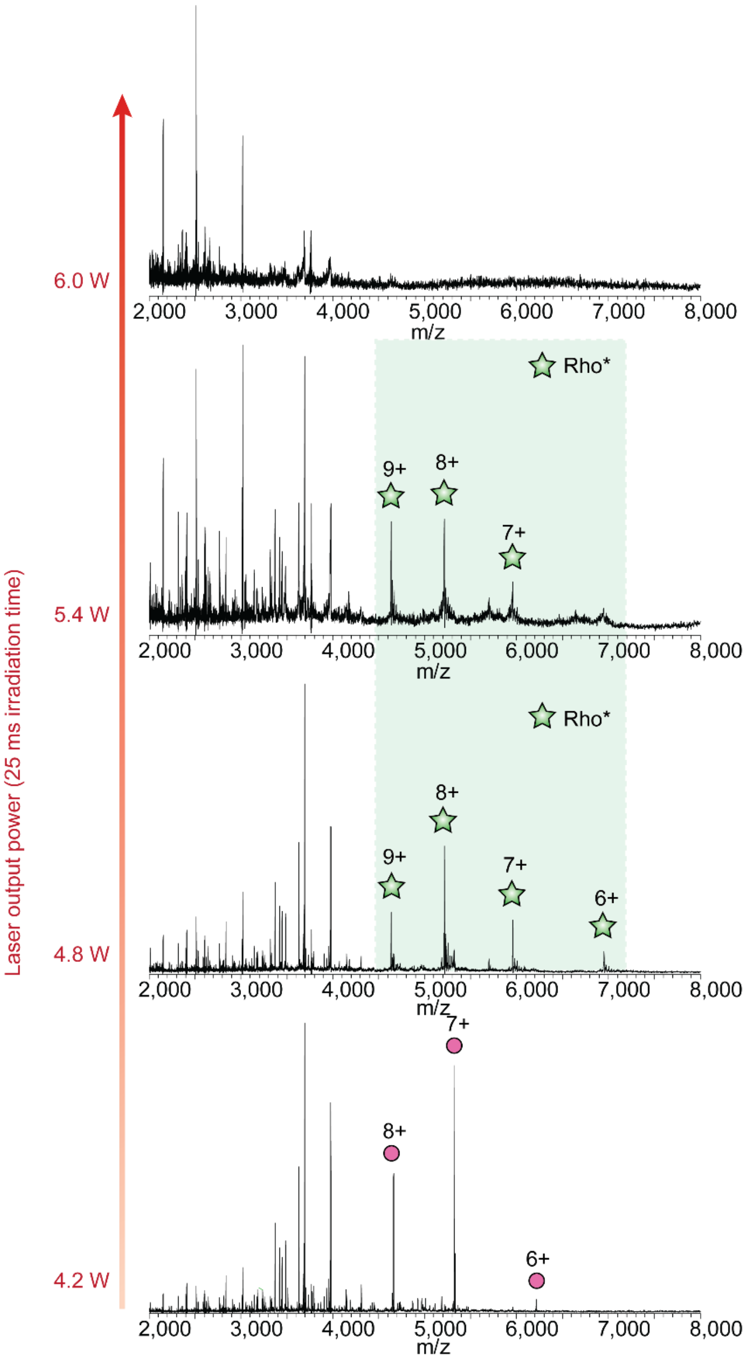

Figure S1. Effect of laser output power on the liberation of membrane proteins from the bilayer. Laser output power was varied from 6.0 W, 5.4 W, 4.8 W, and 4.2 (top to bottom) with a constant irradiation time of 25 ms. Peaks denoted by stars correspond to rhodopsin which is only visible over a narrow range of laser output powers between ~4.8 W and ~5.4 W.

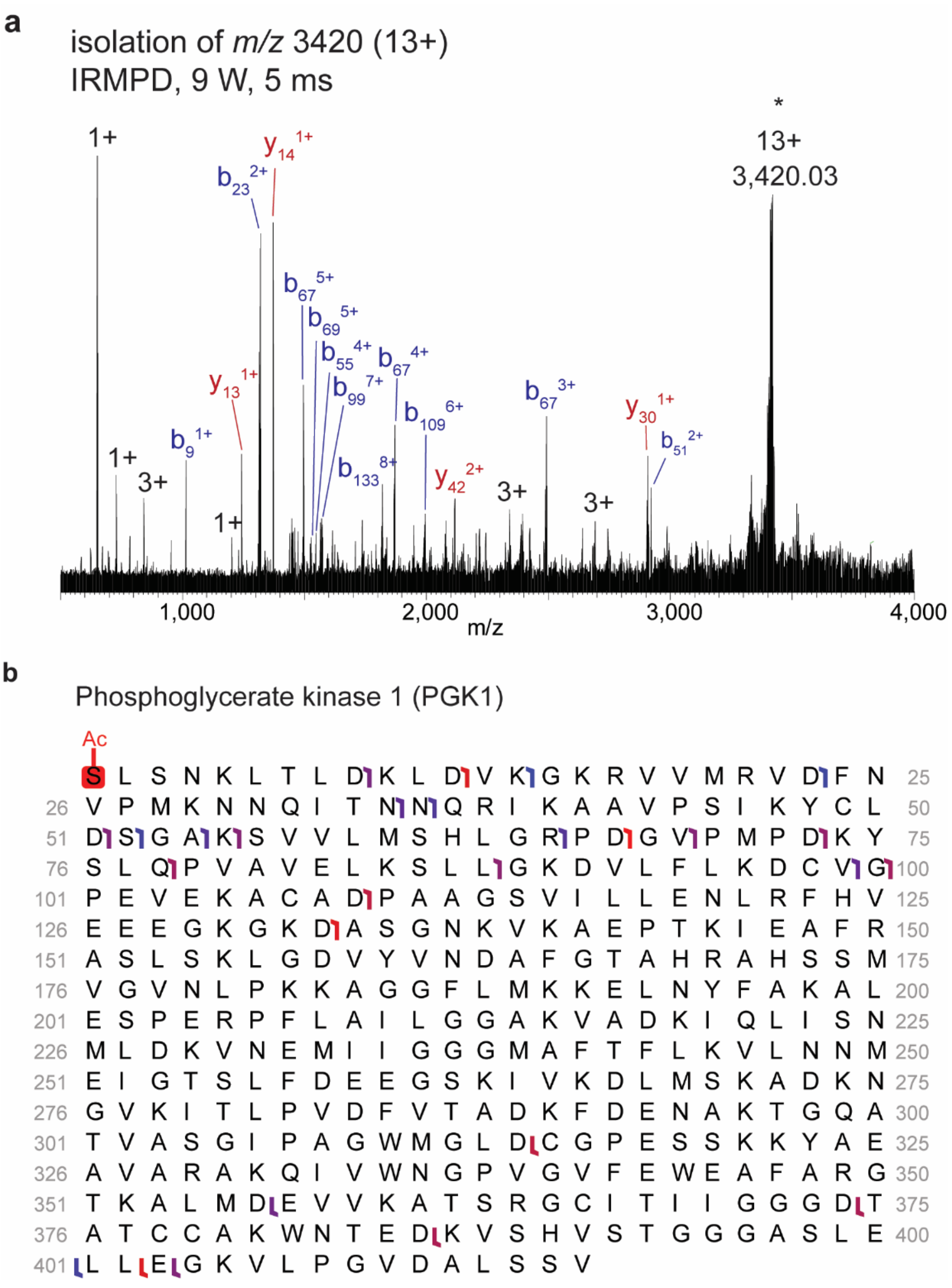

Figure S2. Identification of phosphoglycerate kinase 1 (PGK1). (a) isolation of the 13+ charge state at *m/z* 3,420 with a 35 *m/z* isolation window and subsequent fragmentation via IRMPD (9 W, 5 ms). The MS^2 spectrum shows b-type and y-type ions which correspond to fragment ions originating from with a loss of the initiator methionine and acetylation of serine at position 2. (b) Location of the fragments depicted in a graphical fragmentation map of the protein sequence. Overall, 6% sequence coverage was obtained which was sufficient for protein identification.

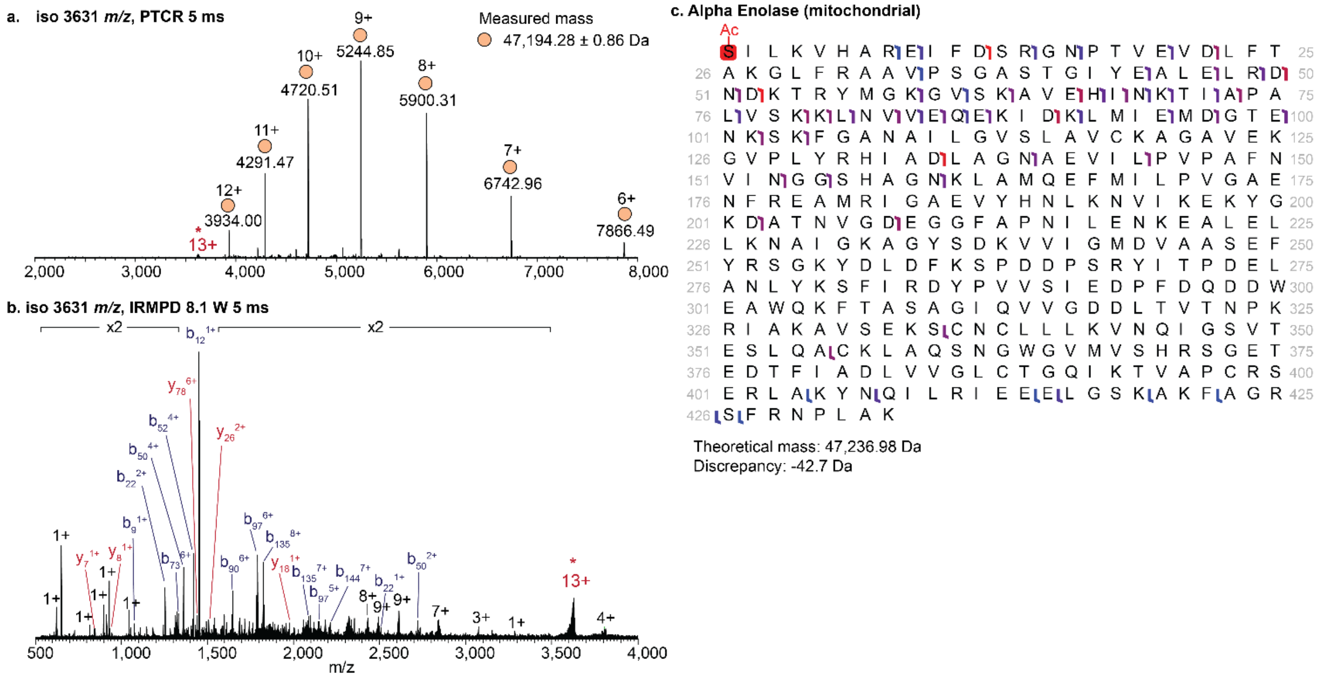

Figure S3. Identification of alpha enolase (ENOA). Isolation of the 13+ charge state at *m/z* 3,631 subjected to (a) a proton-transfer charge reduction (PTCR) reaction to obtain an accurate mass measurement of 47,194.28 ± 0.86 Da and (b) fragmentation via IRMPD (8.1 W, 5 ms). The MS^2 spectrum contained b-type and y-type ions which corresponded to fragment ions originating from alpha enolase (ENOA) with a loss of the initiator methionine and acetylation of serine at position 2. (c) Location of the fragments depicted in a graphical fragmentation map of the protein sequence. Overall, 13% sequence coverage was obtained.

*Mass discrepancy: The measured mass is lower than the theoretical mass by -42 Da. However, there is no evidence for the non-acetylated form in the fragmentation pattern and match fragments at both N- and C-termini which suggest the sequences in those regions are correct. The discrepancy must come from a region in the sequence that is not adequately covered using TDMS; The possible sources of this discrepancy could be a single amino acid variant of one of the following: Arg→Asn, Leu/Ile→Ala, Val→Gly, and Glu→Ser, however this cannot be determined with the sequence coverage provided.

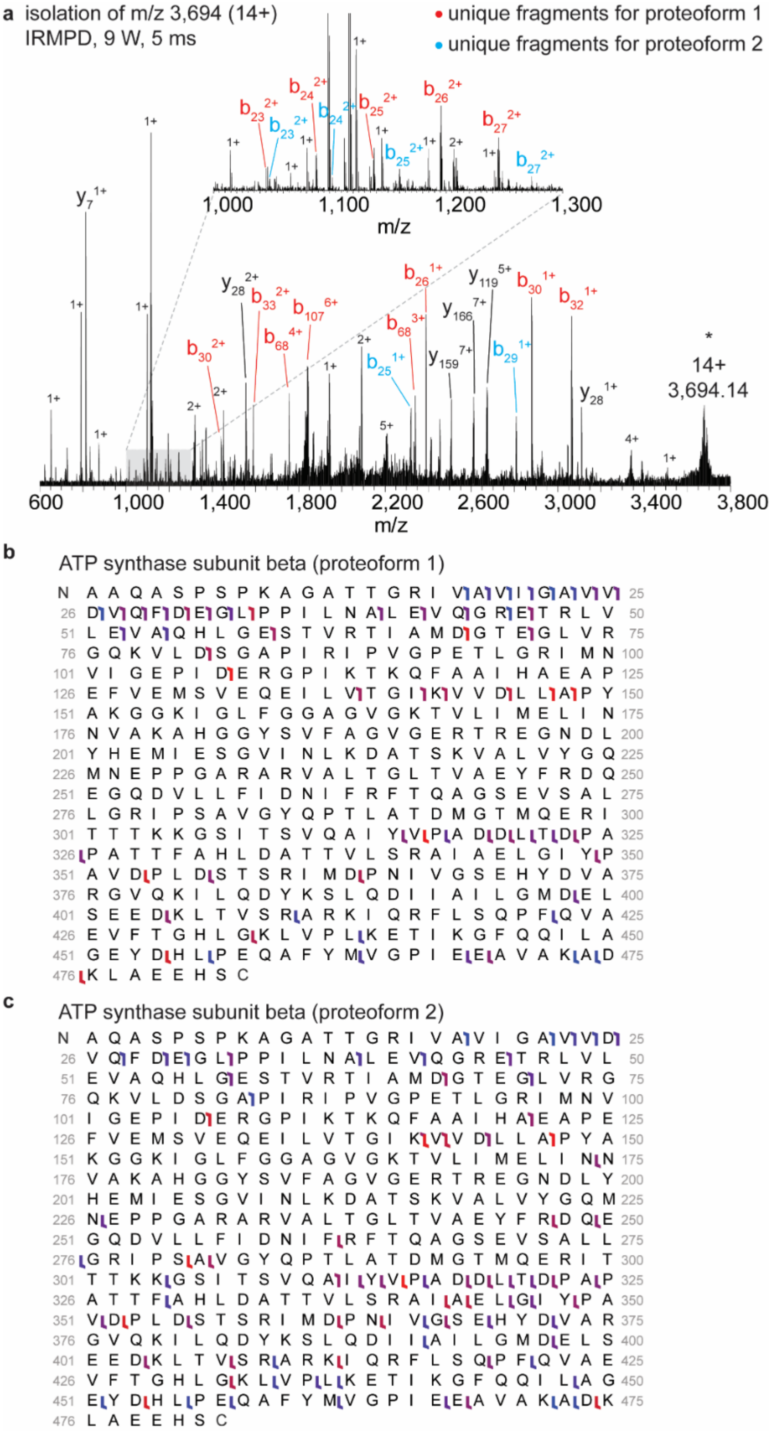

Figure S4. Identification of two forms of ATP synthase subunit beta. (a) Isolation of the 14+ charge state at *m/z* 3,694 and subsequent fragmentation via IRMPD (9 W, 5 ms). A second proteoform at *m/z* 3,688 was co-isolated within the 25 Da isolation window. The resulting MS^2 spectrum contained b-type and y-type ions which corresponded to two different proteoforms of ATP synthase subunit beta. (b) The location of the fragments depicted in a graphical fragmentation map of the protein sequence missing the transit peptide from residues 1 to 46. (c) The location of the fragments depicted in a graphical fragmentation map of the protein sequence missing the transit peptide (1-46) as well as an additional alanine at the N-terminus.

a. iso *m/z* 4150, IRMPD 9 W 5 ms

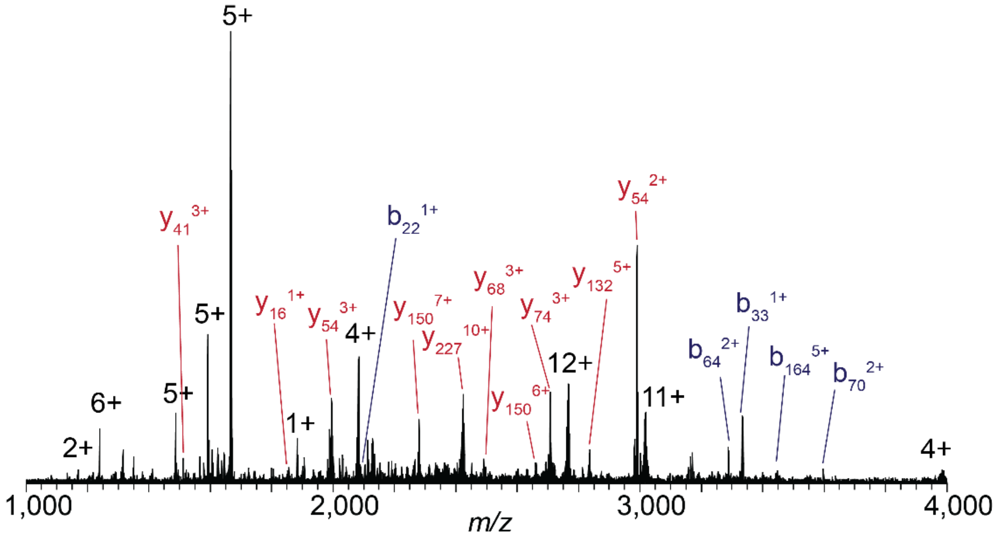

b. Malate Dehydrogenase (mitochondrial)

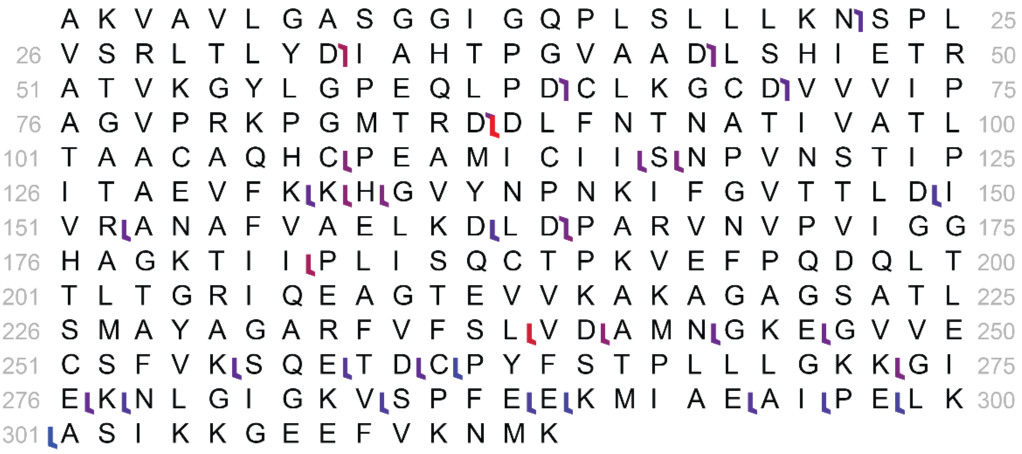

Figure S5. Identification of malate dehydrogenase. (a) Isolation of the 16+ charge state at *m/z* 4,150 with an isolation width of 35 *m/z* and subsequent fragmentation via IRMPD (9 W, 5 ms). The MS^2 spectrum contained b-type and y-type ions which corresponded to fragment ions originating from malate dehydrogenase (mitochondrial). (b) The location of the fragments depicted in a graphical fragmentation map of the protein sequence following the loss of the 24-residue transit peptide at the N-terminus. Overall, 11% sequence coverage was obtained which was sufficient for protein identification.

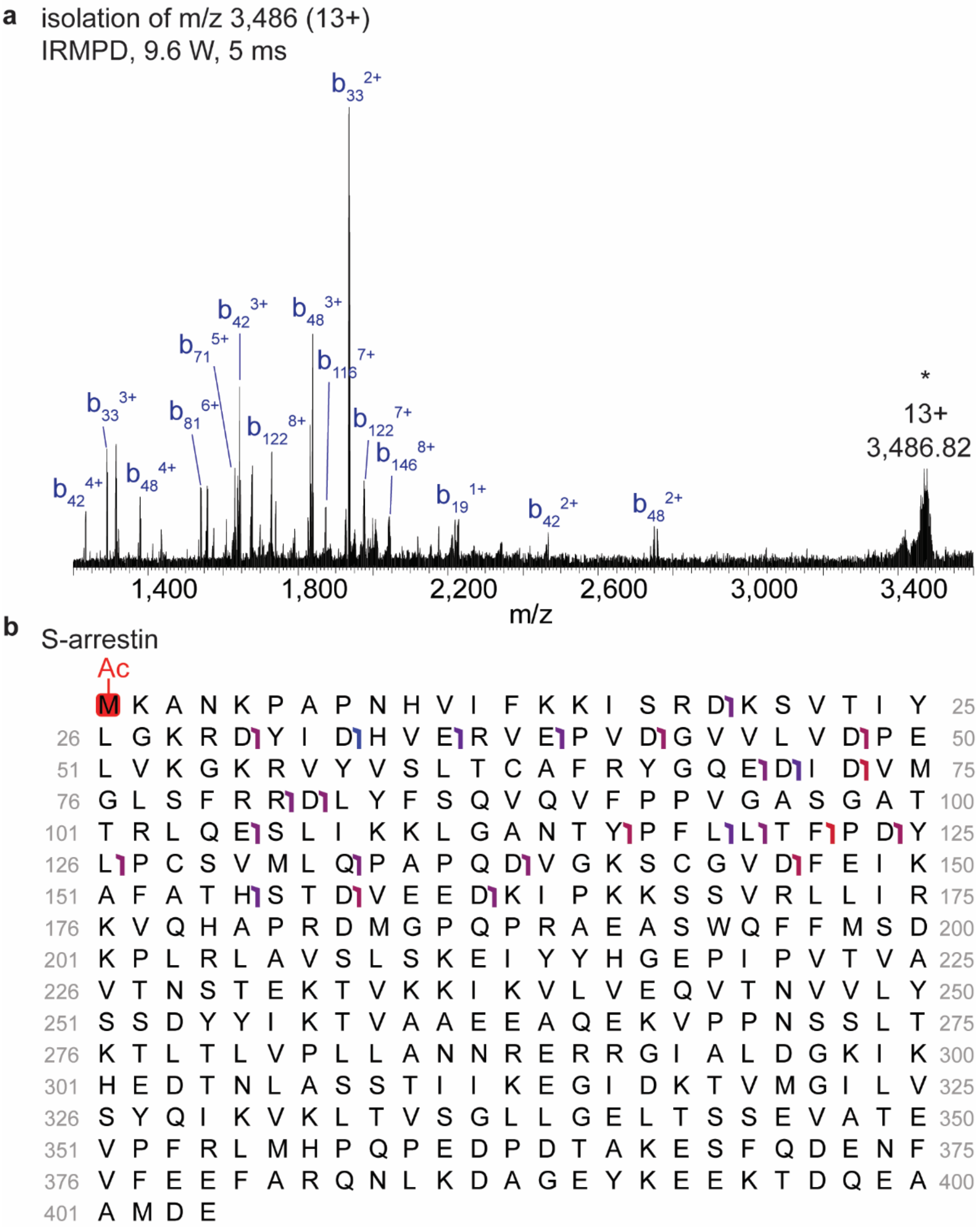

Figure S6. Identification of S-arrestin. (a) Isolation of the 13+ charge state at *m/z* 3,486 and subsequent fragmentation via IRMPD (9 W, 5 ms). The MS^2 spectrum contained b-type and y-type ions which corresponded to fragment ions originating from S-arrestin. (b) The location of the fragments depicted in a graphical fragmentation map of the protein sequence with N-terminal acetylation. Overall, 6% sequence coverage was obtained.

Table S2. Glycopeptides detected by glycoproteomics. Peptide sequence, glycan composition, mass, amino acid position, extracted ion chromatogram (XIC) peak area, and Byonic score. Byonic scores represent the quality of the peptide-spectrum match and range from 0 to 1000, where > 300 represents a good score.

Sequence	Glycans	Mod. Summary	Var. Pos. Protein	XIC area summed	Score
M.nGTEGPNFY.V	HexNAc(3)Hex(3)	N1(NGlycan/1095.3966)	2	1.53E+06	56.26
**Sequence**	**Glycans**	**Mod. Summary**	**Var. Pos. Protein**	**XIC area summed**	**Score**
F.SnKTGVVRSPF.E	HexNAc(3)Hex(3)	N2(NGlycan/1095.3966)	15	2.77E+09	725.43
F.SnKTGVVRSPF.E	HexNAc(3)Hex(3)	N2(NGlycan/1095.3966)	15	2.72E+09	558.05
F.SnKTGVVRSPF.E	HexNAc(3)Hex(5)	N2(NGlycan/1419.5022)	15	4.16E+08	522.6
F.SnKTGVVRSPF.E	HexNAc(3)Hex(4)	N2(NGlycan/1257.4494)	15	3.72E+08	673.82
F.SnKTGVVRSPF.E	HexNAc(3)Hex(4)	N2(NGlycan/1257.4494)	15	3.44E+08	367.06
F.SnKTGVVRSPF.E	HexNAc(3)Hex(6)NeuAc(1)	N2(NGlycan/1872.6505)	15	2.92E+08	575.39
F.SnKTGVVRSPF.E	HexNAc(4)Hex(4)NeuAc(1)	N2(NGlycan/1751.6242)	15	4.91E+07	315.22
F.SnKTGVVRSPF.E	HexNAc(4)Hex(4)	N2(NGlycan/1460.5288)	15	3.78E+07	315.77
F.SnKTGVVRSPF.E	HexNAc(4)Hex(7)NeuAc(1)	N2(NGlycan/2237.7827)	15	2.32E+07	338.22
F.SnKTGVVRSPF.E	HexNAc(2)Hex(3)	N2(NGlycan/892.3172)	15	7.34E+06	382.13
F.SnKTGVVRSPF.E	HexNAc(3)Hex(3)	N2(NGlycan/1095.3966)	15	6.93E+06	316.12
F.SnKTGVVRSPF.E	HexNAc(2)Hex(5)	N2(NGlycan/1216.4229)	15	6.12E+06	312.67

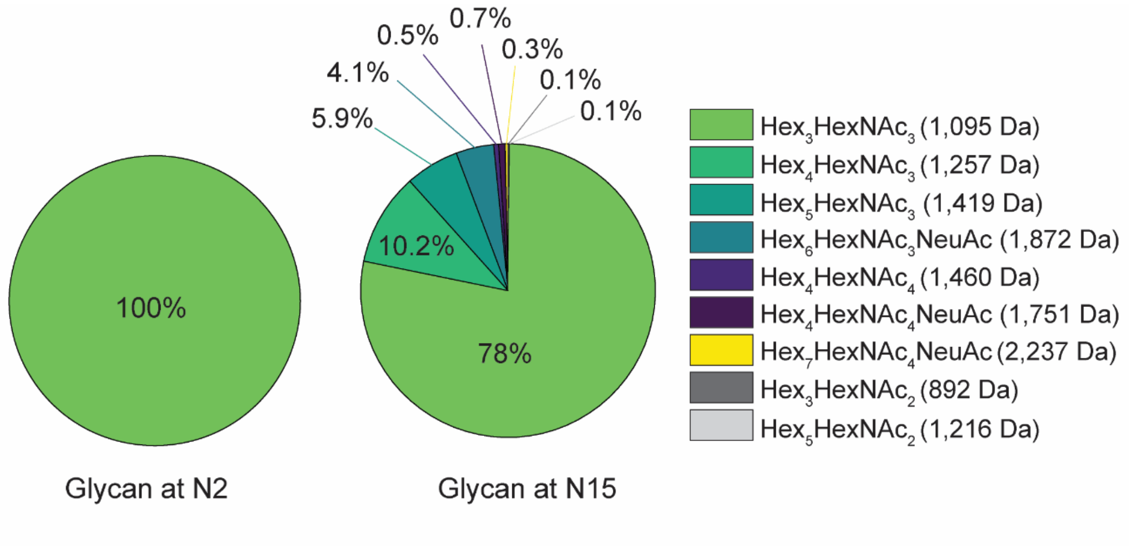

Figure S7. N-glycan composition of rhodopsin. Pie charts representing the relative abundances of glycans at each N-linked site detected by glycoproteomics. The glycan at N2 is fixed as $Hex_3HexNAc_3$ while there is high variability in the glycan composition at N15 (see Table S2).

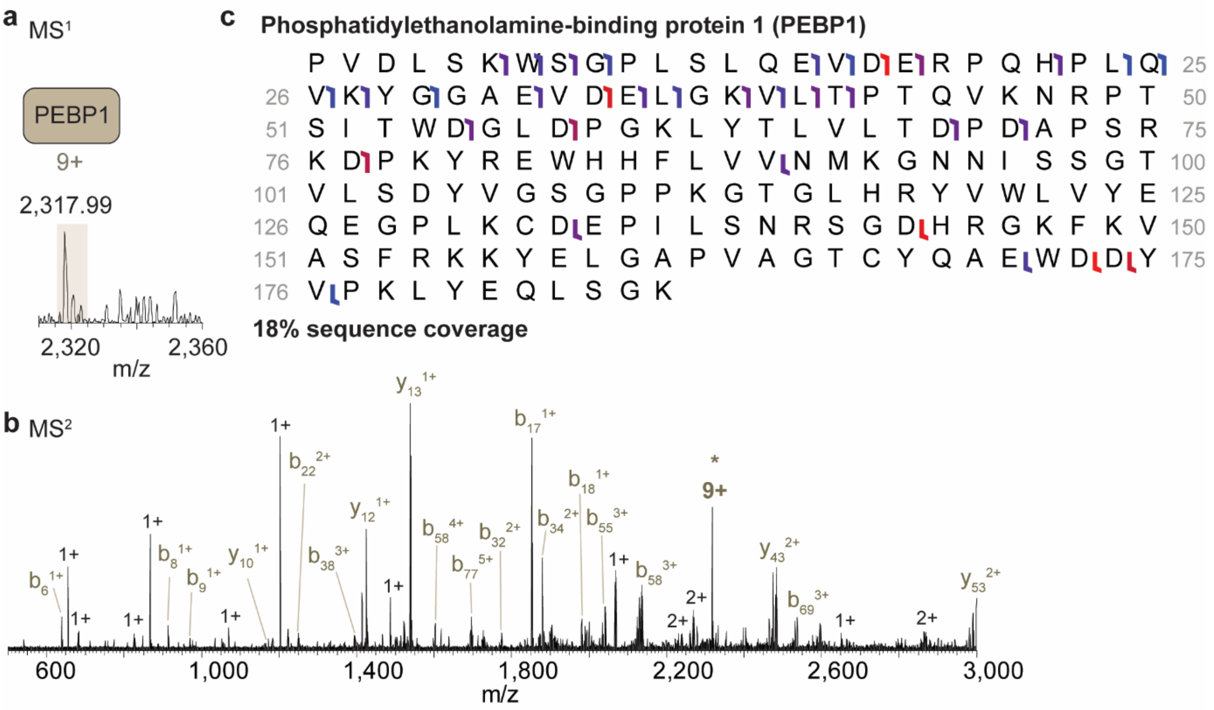

Figure S8. Identification of phosphatidylethanolamine-binding protein 1. (a) Isolation of the 9+ charge state at *m/z* 2317 with an isolation width of 35 *m/z*. (b) The MS^2 spectrum contained b-type and y-type ions which corresponded to fragment ions originating from phosphatidylethanolamine-binding protein 1 (PEBP1). (c) Graphical fragment map showing fragments mapped to onto the protein sequence without the initiator methionine. Overall, 18% sequence coverage was obtained.

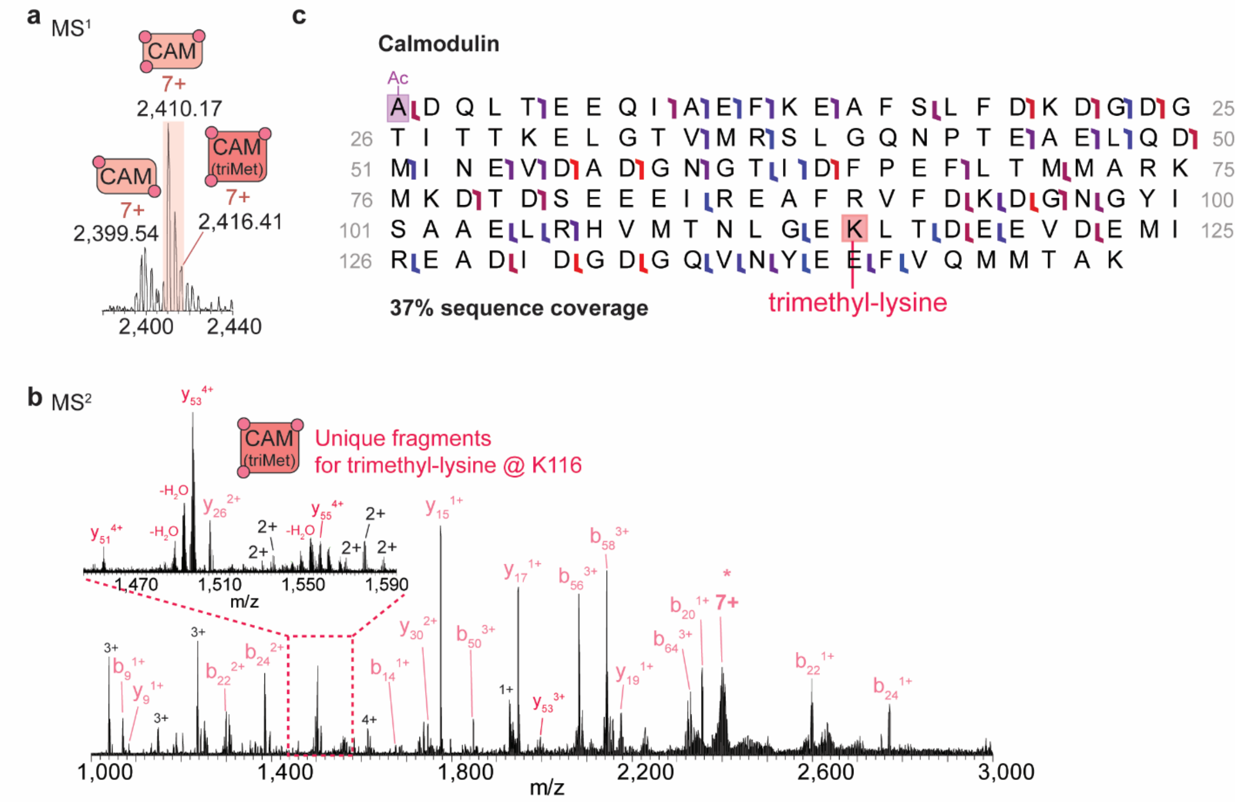

Figure S9. Identification of calmodulin. (a) Isolation of the 6+ charge state at *m/z* 2410 with an isolation width of 35 *m/z*. (b) The MS^2 spectrum contained b-type and y-type ions which corresponded to fragment ions originating from calmodulin (CAM). (c) Graphical fragment map showing the fragments mapped onto the protein sequence without the initiator methionine and N-terminal acetylation of A2. Evidence for trimethylated CAM is embedded within the spectrum in (b). Overall, 37% sequence coverage was obtained.

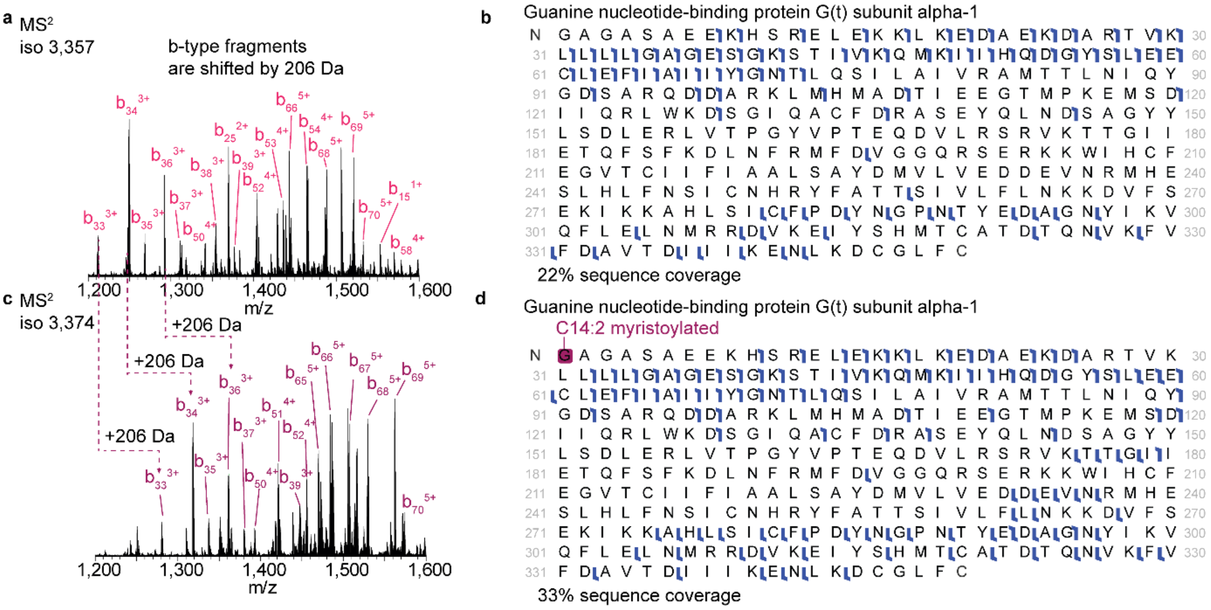

Figure S10. Identification of two proteoforms of Gα. (a) Isolation and fragmentation by IRMPD (8.4 W, 10 ms) of the peak at *m/z* 3,357 produced MS^2 spectra with fragments which corresponded to guanine nucleotide-binding protein subunit alpha (Gα). (b) The location of the fragments depicted in a graphical fragmentation map of the protein sequence missing the initiator methionine. 22% sequence coverage is obtained. (c) Isolation and fragmentation by IRMPD (8.4 W, 10 ms) of the peak at *m/z* 3,374 produced MS^2 spectra with fragments which also corresponded to Gα. (d) The location of the fragments depicted in a graphical fragmentation map of the protein sequence which harbours C14:2 myristoylation at G2 following loss of the initiator methionine. This modification is reflected in the 206 Da mass shift of all b-type fragment ions. Overall, 33% sequence coverage is obtained.

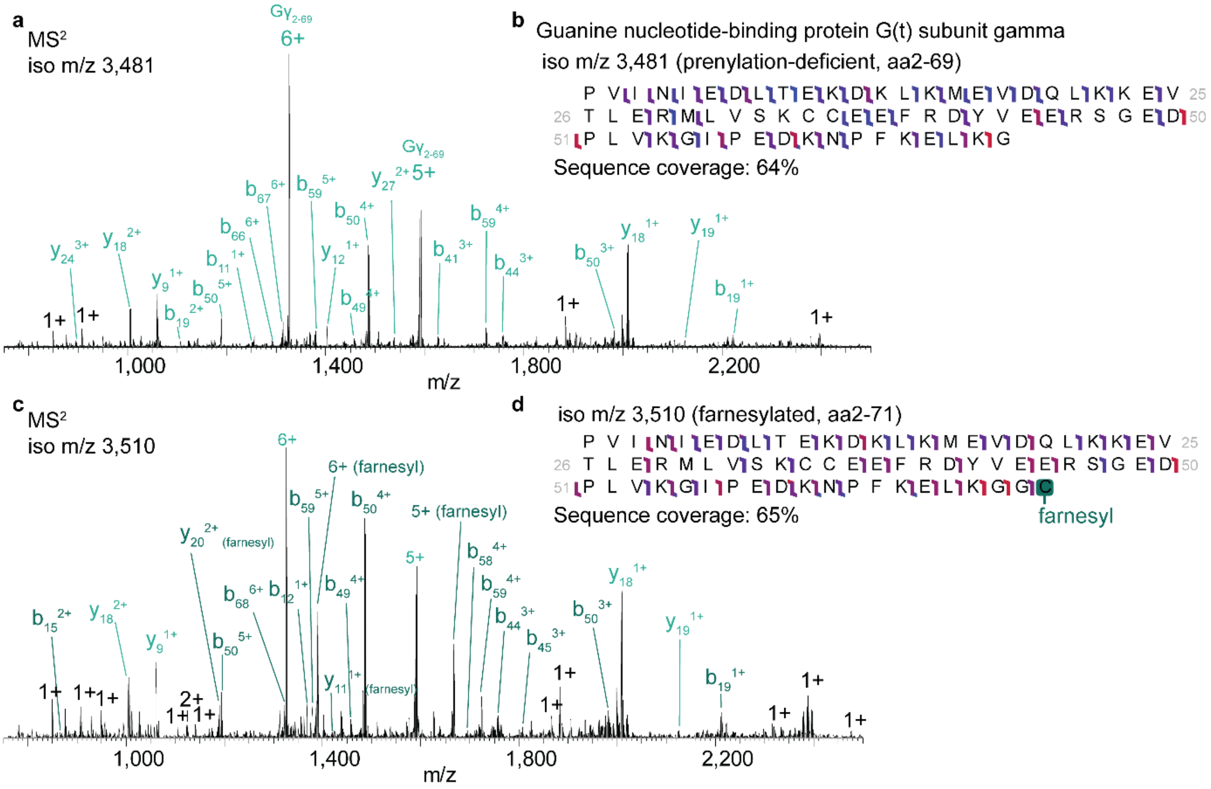

Figure S11. Identification of two proteoforms of GβGγ heterodimers. (a) Isolation and fragmentation by IRMPD (8.4 W, 10 ms) of the peak at *m/z* 3,481 produced MS^2 spectra with fragments which corresponded to guanine nucleotide-binding protein subunit gamma (Gγ). (b) The location of the fragments depicted in a graphical fragmentation map of the protein sequence missing the initiator methionine and truncated at G69 (prenylation-deficient). 64% sequence coverage was obtained. (c) Isolation and fragmentation by IRMPD (8.4 W, 10 ms) of the peak at *m/z* 3,510 produced MS^2 spectra with fragments which also corresponded to Gγ. (d) The location of the fragments depicted in a graphical fragmentation map of the protein sequence which harbours farnesylation at C71. Overall, 65% sequence coverage is obtained.

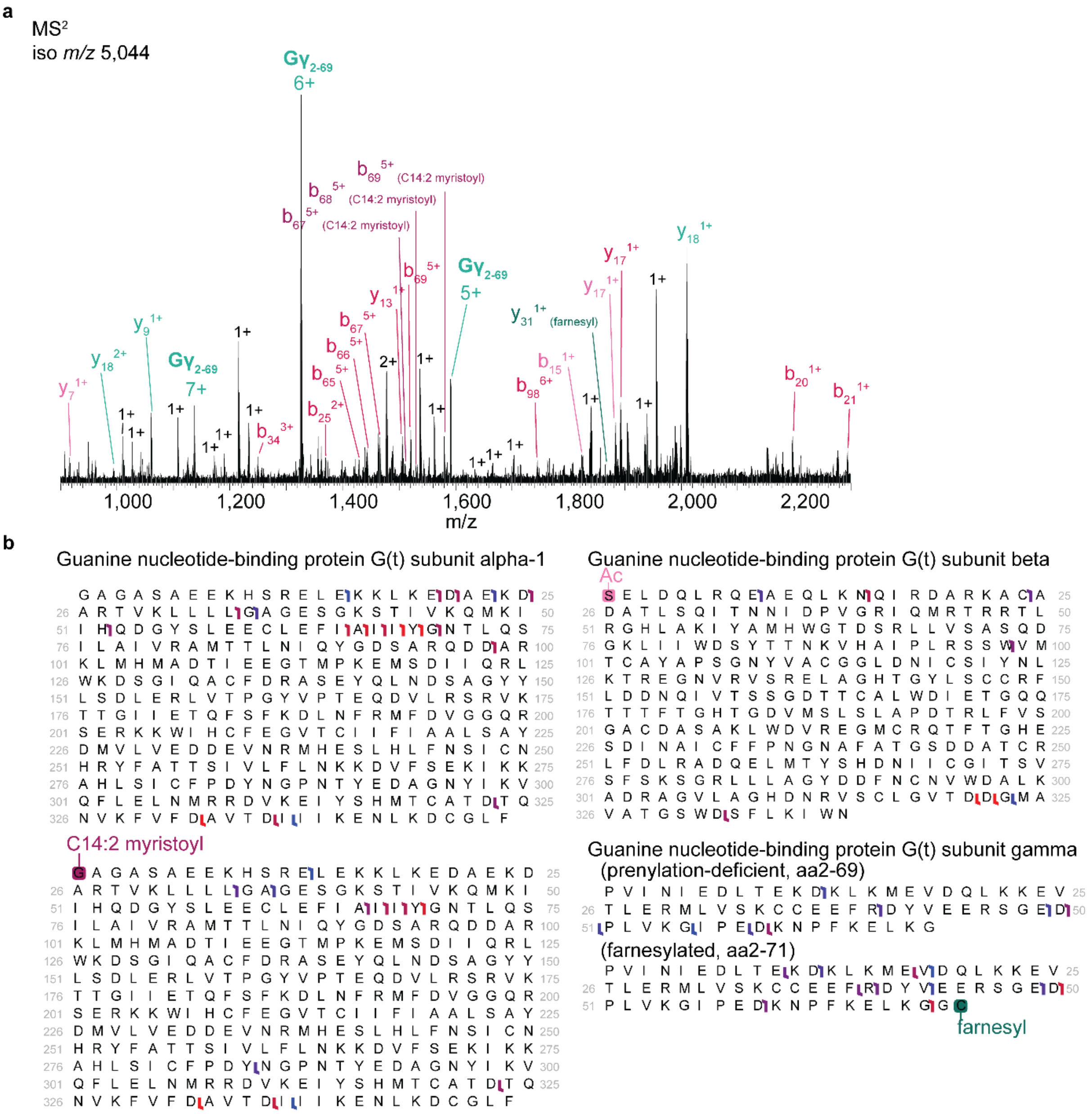

Figure S12. Identification of G protein subunits of the transducin complex. (a) Isolation of the peak at *m/z* 5,044 followed by fragmentation by IRMPD (8.4 W, 10 ms) produced MS^2 spectra with fragments which corresponded to all three proteins present in the heterotrimeric Gαβγ complex (transducin). (b) The location of the fragments depicted in graphical fragmentation maps of the protein sequences for the detected proteoforms of Gα, Gβ, and Gγ.

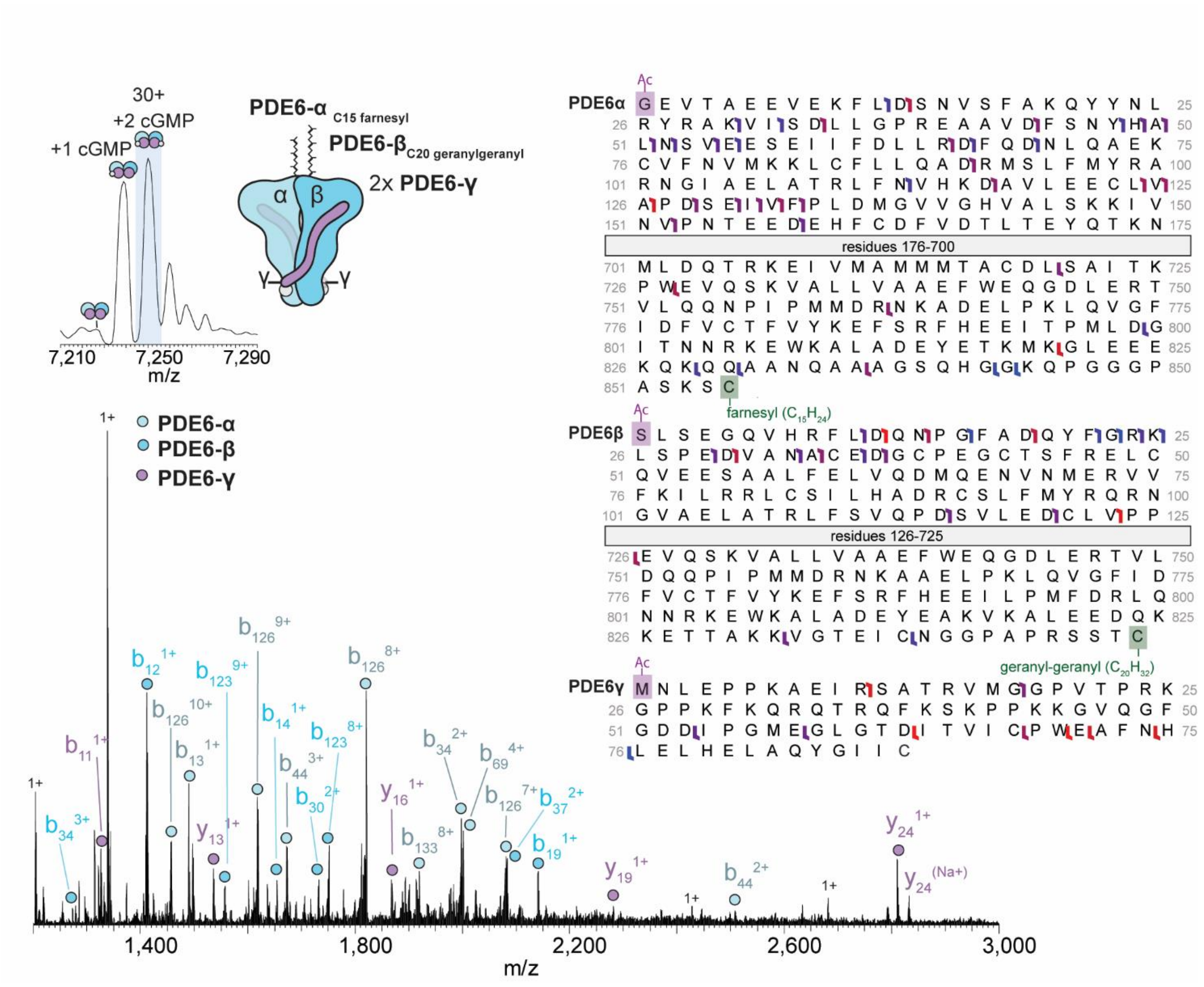

Figure S13. Identification of phosphodiesterase 6 (PDE6). Isolation and IRMPD fragmentation (8.4 W 10 ms) of PDE6 heterocomplex complex at *m/z* 7,250. The MS^2 spectrum reveals fragments for three proteins present in the heterocomplex. We observe fragments which harbour lipidation for PDE6α (farnesylation) and PDE6β (geranyl-geranylation). Inset shows the graphical fragment maps showing the fragments mapped onto the protein sequences for PDE6α, PDE6β, and PDE6γ. Residues 176-700 of PDE6α and 126-725 of PDE6β are omitted as we do not observe fragmentation in those regions.

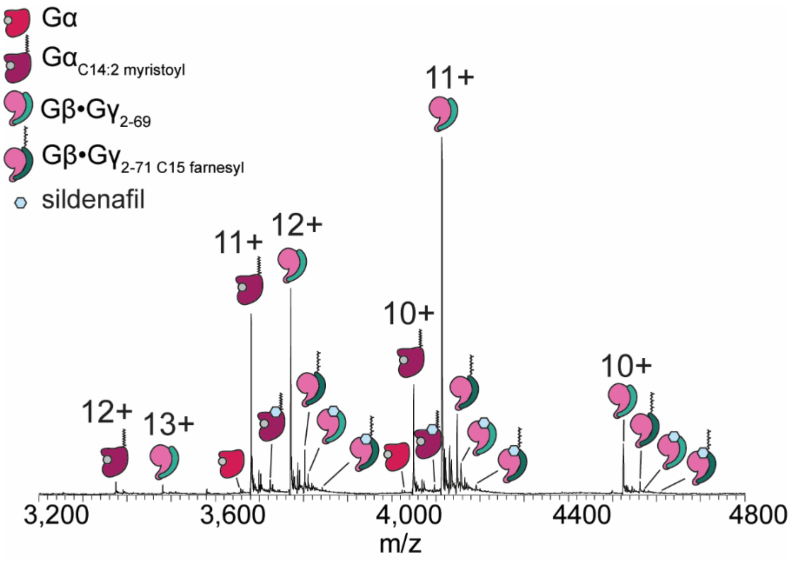

Figure S14. Sildenafil binds to G proteins. Mass spectrum showing evidence for additional off-target binding of sildenafil to G proteins in the intradiscal fraction incubated with 20 µM drug.

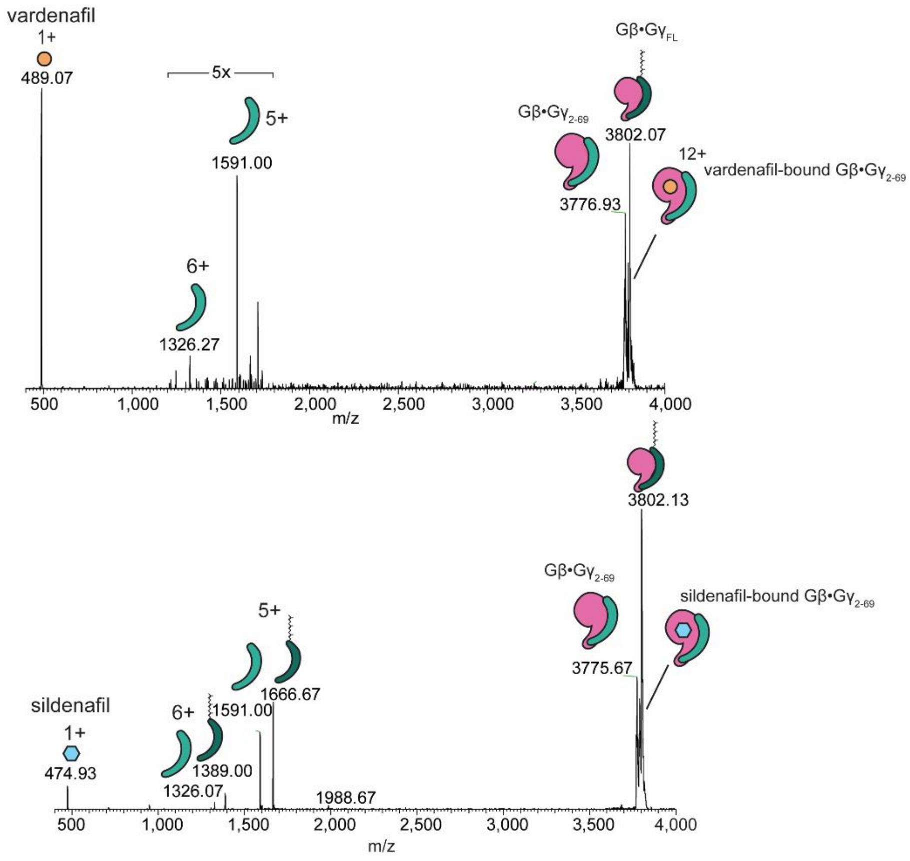

Figure S15. Release of drugs bound to Gβγ heterodimers. Peaks corresponding to drug bound states of Gβγ were isolated and subjected to low-energy resonance collision-induced dissociation (CID) to release the drug bound from the complex. CID effectively liberates the vardenafil (top) and sildenafil (bottom) from the Gβγ complex. Loss of the Gγ subunit is also observed in both spectra.
